# Liquid crystal-powered Mie resonators for electrically tunable photorealistic color gradients and dark blacks

**DOI:** 10.1038/s41377-022-00806-8

**Published:** 2022-04-29

**Authors:** Trevon Badloe, Joohoon Kim, Inki Kim, Won-Sik Kim, Wook Sung Kim, Young-Ki Kim, Junsuk Rho

**Affiliations:** 1 Department of Mechanical Engineering, Pohang University of Science and Technology (POSTECH), Pohang, 37673 Republic of Korea; 2 Department of Biophysics, Institute of Quantum Biophysics, Sungkyunkwan University, Suwon, 16419 Republic of Korea; 3 Department of Intelligent Precision Healthcare Convergence, Sungkyunkwan University, Suwon, 16419 Republic of Korea; 4 Department of Chemical Engineering, Pohang University of Science and Technology (POSTECH), Pohang, 37673 Republic of Korea; 5 Department of Electrical Engineering, Pohang University of Science and Technology (POSTECH), Pohang, 37673 Republic of Korea; 6 POSCO-POSTECH-RIST Convergence Research Center for Flat Optics and Metaphotonics, Pohang, 37673 Republic of Korea; 7 National Institute of Nanomaterials Technology (NINT), Pohang, 37673 Republic of Korea

**Keywords:** Metamaterials, Liquid crystals, Nanophotonics and plasmonics, Micro-optics

## Abstract

Taking inspiration from beautiful colors in nature, structural colors produced from nanostructured metasurfaces have shown great promise as a platform for bright, highly saturated, and high-resolution colors. Both plasmonic and dielectric materials have been employed to produce static colors that fulfil the required criteria for high-performance color printing, however, for practical applications in dynamic situations, a form of tunability is desirable. Combinations of the additive color palette of red, green, and blue enable the expression of further colors beyond the three primary colors, while the simultaneous intensity modulation allows access to the full color gamut. Here, we demonstrate an electrically tunable metasurface that can represent saturated red, green, and blue pixels that can be dynamically and continuously controlled between on and off states using liquid crystals. We use this to experimentally realize ultrahigh-resolution color printing, active multicolor cryptographic applications, and tunable pixels toward high-performance full-color reflective displays.

## Abstract (web summary)

Ellipsoidal-shaped Mie resonators are integrated with a liquid crystal cell to demonstrate electrically tunable reflective structural color metasurfaces that can be modulated continuously from bright spectral colors to dark blacks.

## Graphical abstract

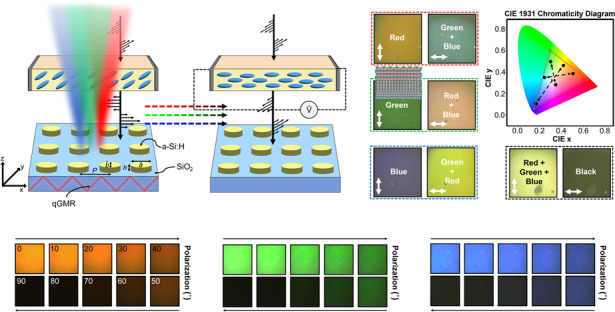

## Introduction

Metasurfaces consisting of meticulously designed subwavelength structures, known as meta-atoms, have been actively researched as optical components to modulate electromagnetic (EM) waves^1^. Various practical applications of metasurfaces have already been demonstrated such as in holography^2–4^, lensing^4–8^, and all-solid-state lidar^9,10^. By utilizing nanoscale structures, the operating wavelengths of metasurfaces are moved to the visible regime, thus providing a platform for high-resolution and -performance nano-optics with an extremely small form factor. Upon continuous advances in nanofabrication techniques^11^, commercially viable metasurfaces are becoming a reality. Along with controlling the wavefront of light for focusing and imaging with metalenses, display technology using metaholography or advanced color filters is another promising application of metasurfaces that could have profound impacts in many fields such as direct view displays, image projection systems, and even micro-displays for augmented reality (AR) headsets.

Taking inspiration from nature, structural coloration is a method of reflecting or transmitting certain wavelengths of light. Compared to traditional methods of producing aesthetically pleasing colors through pigments and dyes, structural colors based on metasurfaces have the additional benefits of being resistant to fading over time and providing ultrahigh-resolution, all while also potentially being environmentally friendly^12^ with applications in optical security^13,14^ and biosensing^15^. Notably, metasurfaces have been exploited as a method of increasing the color performance and resolution of organic light emitting diode-based displays to over 10,000 pixels per inch (PPI)^16^. Although structural colors using various plasmonic^17^ and dielectric materials^18–22^ have been proven, with the gamuts that extend beyond conventional display technologies, their static nature is a fundamentally limiting factor. In the current Internet of things (IoT) era, dynamically tunable metasurfaces and colors would be a giant step forward in the incorporation of such planar optical components into consumer devices.

Recently, research into actively tunable structural colors using metasurfaces has been plentiful, with a number of different approaches^23–25^. Materials with tunable optical properties, such as phase change materials^26–28^, or through chemical reactions^29^ have been demonstrated. However, these approaches generally rely on the use of plasmonics, which comes with the drawback of substantial Ohmic losses in the visible region, which detrimentally affects the brightness and color performance. Other noteworthy strategies include employing electrochromic semi-conductors^30–32^ or polymers^33^ with optical properties that can be controlled electrically, and also the physical manipulation of the spatial locations of the meta-atoms through the use of stretchable substrates^34^.

In conventional displays, liquid crystals (LCs) have been a key component in color rendition for a number of years. Their versatility has also led to numerous applications in combination with metasurfaces for electrically tunable active nanophotonic devices^35–39^, including examples of structural color^40–47^. Despite these efforts, for active metasurface-based structural color, several functionalities still need to be realized simultaneously. Namely, bright, saturated colors are required, along with a dark black state. Furthermore, the management of intermittent states between bright and dark is paramount to allow the production of gradients of color to produce photorealistic results through access to the entire color gamut. Up to now, these functionalities have yet to be realized in a single tunable metasurface. Moreover, examples of tunable structural color using LCs have relied on plasmonic metasurfaces, which suffer from unavoidable Ohmic losses at blue wavelengths, limiting their applicability at those wavelengths, and black states have only been achieved through the use of a secondary polarizer to block the polarized light after interaction with the metasurface. More details of comparisons with our work and previously reported tunable structural color metasurfaces with LCs can be found in Supplementary Note 1.

**Fig. 1:**
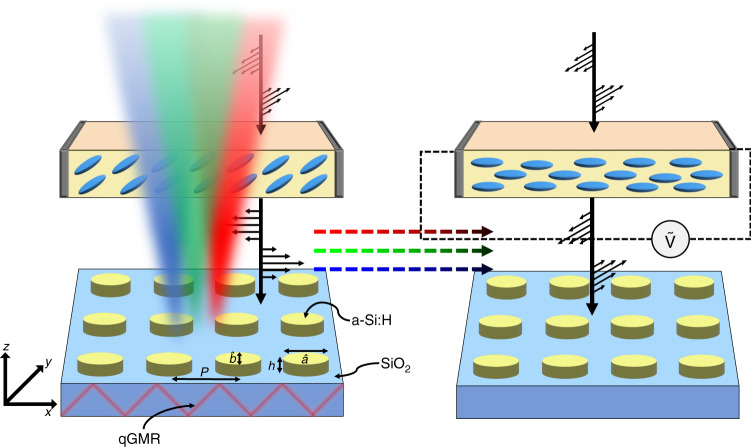
Elliptical meta-atoms combined with LCs for electrically tunable structural color. Schematic of the elliptical meta-atoms combined with an LC cell for electrically tunable color, and its working mechanism through the enhancement of Mie scattering through the quasi-guided mode resonances (qGMR) induced from the lattice. Electrical tuning of the incident polarization of light via an input bias (Ṽ) on the LC cell allows for the continuous tuning of the scattering power of the meta-atoms, which manifests a modulation in the color production from bright vivid colors to dark blacks

Here, we numerically design and experimentally demonstrate electrically tunable all-dielectric metasurfaces and investigate their use in cryptographic applications, as well as for full-color reflective displays. The ellipsoidal meta-atoms provide a polarization dependent optical response that is modulated through the implementation of an electrically tunable LC cell, providing an almost linear transition between bright saturated ‘on’ states and dark black ‘off’ states (Fig. 1). We prove the ability of the metasurfaces to not only produce high-quality photorealistic color prints, and as a technology to hide and display multicolored information, but also as fully controllable color pixels without the need for any additional optical elements, such as analyzers. Furthermore, the underlying principles of the working mechanisms are revealed and discussed in terms of Mie scattering and the hybridization with quasi-guided mode resonances (qGMR) that originate from the lattice. This work has the potential to be extended to fully tunable reflective displays that cover the whole range of different hues with controllable brightness, low power film displays that are only activated under appropriate security conditions, and micro-displays for AR headsets, having impact not only on the display industry but also with promise for application in security systems.

## Results and discussion

**Fig. 2:**
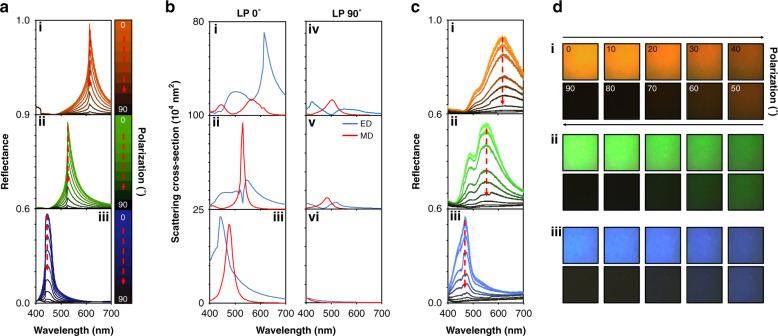
Simulated reflectance and scattering cross-sections of the elliptical meta-atoms, and experimental results. **a** Simulated reflectance spectra of the polarization sensitive meta-atoms for (i) red, (ii) green, and (iii) blue colors with the incident light of linear polarization (LP) from along the long axis *â* (LP 0˚) to along the short axis *b̂* of the meta-atom (LP 90˚). As LP is modulated, the reflectance is gradually reduced, until a dark state is produced. **b** Calculated scattering cross-sections of the meta-atoms to produce (i) red, (ii) green, and (iii) blue colors under LP 0˚. For red, the electric dipole (ED) is dominant, while for green the magnetic dipole (MD) is dominant, and for blue the ED and MD spectrally overlap. Under LP 90˚, the scattering cross-sections for (iv) red, (v) green, and (vi) blue become almost negligible as quasi-guided modes are no longer supported and the scattering is unenhanced. **c** Measured reflectance spectra for (i) red, (ii) green, and (iii) blue metasurfaces with respect to LP axis from 0 to 90^o^. **d** Corresponding optical micrographs of the 100 × 100 µm (i) red, (ii) green, and (iii) blue metasurfaces. The dotted black lines indicate the location of the Rayleigh-Wood anomaly

One of the simplest strategies to create tunable reflective metasurfaces is to integrate polarization dependent anisotropic meta-atoms with a LC modulator to electrically control the linear polarization (LP) of the incident light. We employ low-loss hydrogenated amorphous silicon (a-Si:H)^48^ for the meta-atoms, designed to have a high refractive index (*n*) and low extinction coefficient (*k*) across the visible spectrum (Supplementary Note 2). This allows us to exploit the strong Mie scattering properties of the meta-atoms to produce bright spectral colors in reflection. We determine that ellipsoidal shaped meta-atoms provide the best reflectance and LP-dependency (Supplementary Note 3). By carefully designing the Mie scattering response of the dielectric meta-atoms and arranging them on a lattice with specific periodicities^20^, the hybridization of Mie scattering of the meta-atoms with the qGMR from the periodic array greatly amplifies the response, resulting in the desired spectral modulation (Supplementary Note 4). This can be understood by considering the meta-atoms playing three distinct roles. Namely: (1) Mie resonators that allow light to be outcoupled to free space through their resonant modes such as the electric and magnetic dipoles (ED and MD), (2) A subwavelength grating through the arrangement of the meta-atoms on a 2D periodic lattice, and (3) A thick waveguiding layer that supports the in-plane propagation modes. The refractive index of the background medium (*n*_back_) and substrate (*n*_sub_), as well as the periodicity (*P*) of the nanostructures are important factors that contribute to the momentum matching conditions to excite the qGMR^49^. The lower bound of these parameters determines the location of the Rayleigh-Wood anomaly, where the normally incident light is diffracted and propagates at 90°, i.e., in the plane of the meta-atoms, and the diffuse anomalies described by Fano are located. Here, we use a glass substrate in the background of air (i.e.*, n*_sub_ = 1.46 and *n*_back_ = 1). This helps to bring the lattice enhanced scattering into the visible regime for use for bright structural color production, as well as being relevant for real-world applications as no extra index matching background layers are required. The upper bound of the qGMR is determined by the effective refractive index of the meta-atom unit-cell, i.e., the geometry of the meta-atom. Therefore, the design of the meta-atom requires a fine balance between all of the different parameters. We note that extra protective layers could be added if *n* of the medium and the dimensions of the meta-atoms are designed accordingly to produce the desired scattering and momentum matching conditions. The height of the meta-atoms is another key parameter for determining how strongly the EM fields can be confined inside the structure through the formation of displace currents along the propagation direction. Therefore, a height of 110 nm is chosen for the meta-atoms. This results in a slight loss of performance in terms of the color gamut that can be produced (Supplementary Note 5). In compensation, however, we can achieve outstanding black states and grayscales of color through the modulation of LP axis (*L̂*) from 0 to 90°, i.e., from along the long axis (*â*) to the short axis (*b̂*) of the meta-atoms (Fig. 2a). Furthermore, despite this sacrifice, the achieved gamut is still comparable with sRGB (Supplementary Note 6), proving the validity for this decision. The optimal designs are chosen with regards to both the color and black states using the definition of color difference (*ΔE*) from the International Commission of Illumination (CIE), which aims to take into account the non-uniformities of the human eye being more sensitive to certain colors compared to others (Supplementary Note 7). The designs that give the lowest total *ΔE* with consideration of the black state (i.e., *ΔE*_*RGB*_ + *ΔE*_*black*_) are therefore determined to be: *P* = 420 nm, *â* = 336 nm, and *b̂* = 118 nm for red; *P* = 360 nm, *â* = 266 nm, *b̂* = 112 nm for green; and *P* = 300 nm, *â* = 204 nm, and *b̂* = 73 nm for blue, respectively. The calculated *ΔE* for the red, green, and blue colors are 15.8, 20.4, and 13.8, respectively, with *ΔE* for the black, off states, of 4.6, 3.5, and 6.9.

We perform multipole analysis to uncover the physical origin of the scattering moments in the anisotropic ellipsoid meta-atoms to produce red, green, and blue colors (Fig. 2b) (Supplementary Note 8). It is clear to see that when *L̂*∥*â*, strong MD and ED responses are excited. When *L̂*⊥*â*, however, the scattering is extremely suppressed as the momentum matching conditions are no longer met, and the dominant scattering modes lie on the blue side of the Rayleigh-Wood anomaly, prohibiting them from being enhanced through the lattice resonances. Interestingly, for the red pixel, the dominant mode is the ED rather than the MD. This can be understood through consideration of the physical dimensions of the meta-atoms that require larger periodicities to enhance the scattering through the qGMR at longer wavelengths. Since the height of all meta-atoms must be consistent due to fabrication limitations, the meta-atoms designed to produce red are therefore rather short and fat with a very low aspect ratio, which hinders the excitation of the MD.

To experimentally demonstrate the LP dependency of the designed colors, we fabricate color swatches for the optimized red, green, and blue designs. Measured reflectance spectra and corresponding optical micrographs of the fabricated 100 × 100 µm samples are shown in Fig. 2c, d. When *L̂* is manipulated from 0 to 90°, we observe the red, green, and blue reflectance to be linearly controllable with remarkably dark black states. We measure the contrast ratio of reflectance to be 42, 37, and 47 for the red, green, and blue metasurfaces, respectively. Although the measured reflectance spectra are in good agreement with the simulations, the peaks of the green and red spectra in particular are broader. These discrepancies are attributed to fabrication errors such as slight inclines in the sidewall and the deviations from the designed elliptical shapes of the meta-atoms which have been shown to hinder such high quality factor resonances^50,51^.

**Fig. 3:**
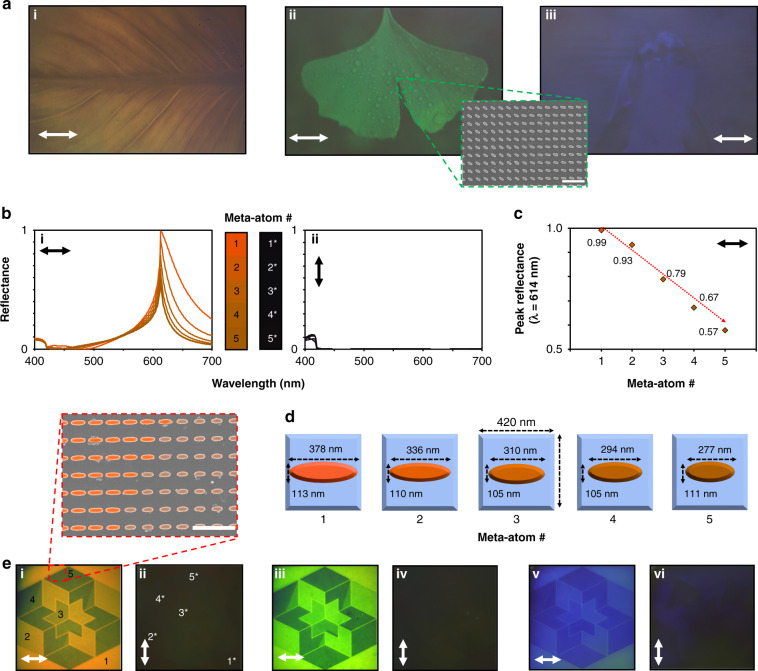
Photorealistic color printing and multicolor cryptography. **a** Detailed color prints under LP 0° for (i) red, (ii) green, and (iii) blue metasurfaces. The original images are discretized into 91 gray levels, and the meta-atoms are rotated with the corresponding angle to produce the desired color. The inset in (ii) shows a scanning electron microscope image, displaying the rotated meta-atoms. The scale bar represents 1 µm. **b** Simulated reflectance spectra of the 5 selected meta-atoms with different geometrical parameters to produce different shades of red while maintaining dark blacks for (i) LP 0° and (ii) LP 90°. **c** Reflectance at the peak wavelength of 614 nm for the five different meta-atoms under LP 0°. **d** Schematic representations of the 5 meta-atoms. **e** Cryptographic color images using meta-atoms with various structural parameters within the same periodicity for (i) *P* = 420 nm (red), (iii) 360 nm (green), and (v) 300 nm (blue). Under LP 0°, multiple shades of the color are produced (i, iii, v), while they are all turned to black and hidden under LP 90° (ii, iv, vi). The inset in (i) shows a faux-colored SEM image, highlighting the meta-atoms with various geometric parameters. The scale bar represents 1 µm. The numbers in (i) and (ii) correspond to the related colors in (**b**). All arrows denote the direction of LP

As a practical presentation of high-resolution color printing based on the designed meta-atoms, we fabricate several metasurfaces to produce photorealistic images (Fig. 3a). Grayscale images are discretized into 91 steps depending on the brightness of the pixel to represent 91 levels of LP (from 0 to 90° in 1° steps), and the relevant meta-atoms are rotated by the necessary angle to achieve the required reflectance. When the ~500 × 300 µm samples are illuminated with LP 0°, the high contrast photorealistic image is clearly displayed. For these single-color microprints, we assume that each pixel is represented by a single meta-atom as the rotation of local meta-atoms determines the scattering properties and therefore color, and *P* is constant over the whole metasurface, akin to the local phase modulation often utilized in geometric phase metaholograms. Since the color depends on *P*, the PPI for the red (Fig. 3a-i), green (Fig. 3a-ii), and blue images (Fig. 3a-iii) are ~60,000, ~70,000, and ~85,000, respectively.

Since the Mie scattering response of the meta-atoms is dependent on their geometry, it is possible to create anisotropic meta-atoms that display different shades and hues of colors for one polarization of light, while still maintaining the dark black states for the orthogonal polarization by designing meta-atoms with different dimensions along *â* and *b̂* of the ellipsoid within the same *P* (Supplementary Note 9). The simulated reflection spectra of the red meta-atoms with *P* = 420 nm producing 5 distinct shades of red are shown in Fig. 3b, while Fig. 3c shows the decrease in the reflectance at the peak wavelength of 614 nm. This is achieved by varying *â* to control the color, while simultaneously modulating *b̂* to achieve the consistent black states (Fig. 3d). We exploit this to demonstrate multicolored cryptographic images that can be switched between on and off states by modulating $\hat L$ from 0 to 90^o^ (Fig. 3e). Each image is made up of 1000 × 1000 meta-atoms, so the samples are 420 × 420 µm, 360 × 360 µm, and 300 × 300 µm in size for the red, green, and blue images, respectively. This technique could be used to hide colorful aesthetic information that can be uncovered when illuminated by the correct LP and completely hidden under the orthogonal LP.

**Fig. 4:**
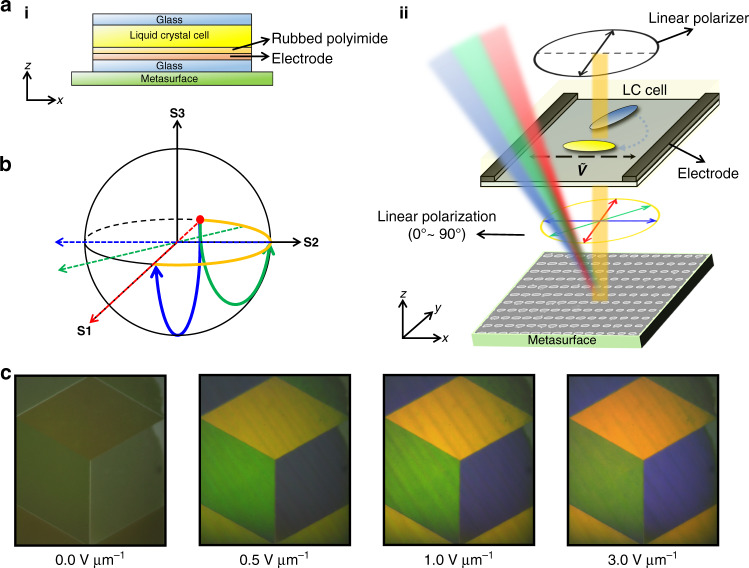
Liquid crystal design and integration. **a** Schematic illustration of the LC integrated metasurface. (ii) Incident LP is modulated through the LC cell. The orientation of LCs at *E* = 0.0 and =3.0 V µm^−1^ is denoted by blue and yellow molecules, respectively. **b** Poincare sphere representing the change in LP state. The solid blue and green lines describe the polarization transitions at *E* = 0.0 and 0.4 V µm^−1^, respectively, with the rotation axis of the Poincare sphere (dotted lines). The orange line indicates the range of LP states that we manipulate. **c** Experimental demonstration of electrically tunable cryptographic images using LC integrated polarization sensitive metasurfaces. The brightness of the image is electrically tuned from dark black to bright colors using an external bias from *E* = 0.0 to 3.0 V µm^−1^

In order to provide electrical tunability to the reflective color metasurfaces, we integrate them with a LC cell. The cell is designed to modulate the incident LP light that illuminates the metasurface. A planar alignment layer is spin-coated onto the top glass substrate (with no electrode) and the bottom substrate with interdigitated ITO electrodes (Fig. 4a). The two substrates are rubbed to provide a tangential orientation to the LCs (i.e., the long axis of the LC molecules is perpendicular to the substrate normal) and then assembled in an anti-parallel fashion with a gap of 5.4 μm. Finally, the cells were filled with a nematic LC, 5CB(4-Cyano-4′-pentylbiphenyl), and attached onto the metasurface (Supplementary Note 10). The incident light first passes through a linear polarizer with its optic axis parallel to the *y*-axis. As the LP 90° then passes through the LC cell, we can precisely control the LP state by controlling the in-plane electric field that rotates the optic axis of the LCs, thus changing the rotation axis in the Poincare sphere (Fig. 4b).

Using the LC integrated metasurfaces, we demonstrate electrically tunable full-color active cryptographic prints. By aligning *L̂* along *â*, the images can be fully displayed and hidden with continuous intermediate states of reflectance by controlling the applied electric field (Supplementary video 1). The fabricated 350 × 400 µm color prints show vivid color rendition and dark states as designed (Fig. 4c). Since the size of the meta-atoms for different colors require different *P*, the size of the image i.e., number of pixels, to cover the same physical space was calculated for each color and combined to form the final image. Slight mismatches between the boundaries of each color led to visible lines where the reflection of the background can be seen through the image, which could be improved by designing the image to have a more precise alignment between colors to alleviate the obvious gaps.

**Fig. 5:**
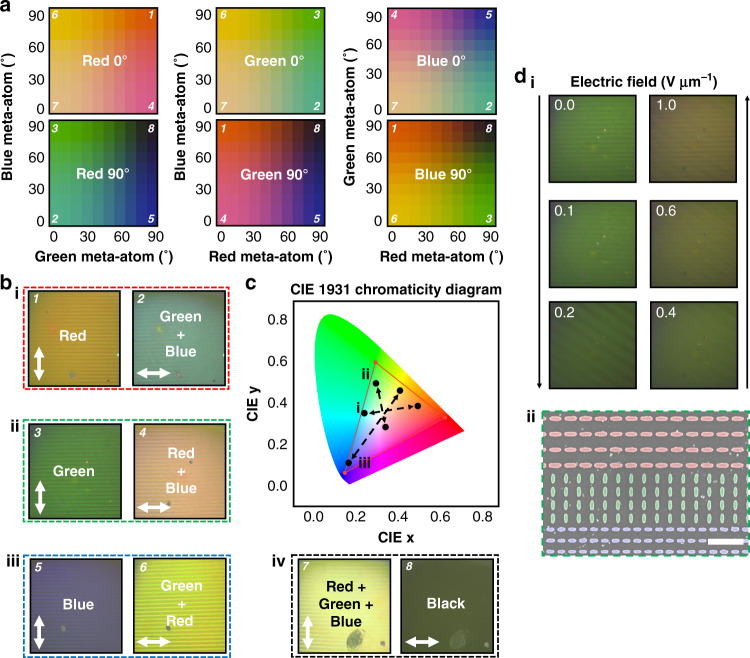
Electrically tunable color pixels for reflective displays. **a** Calculated color palettes by combining the measured reflectance spectra of the red, green, and blue metasurfaces with rotated meta-atoms in a 1:1:1 ratio. **b** Fabricated metasurfaces with rotated subpixels for (i) red, (ii) green, (iii) blue, and (iv) white colors. Under LP 0°, two colors are successfully mixed to produce (i) cyan, (ii) magenta, and (iii) yellow, while under LP 90°, the desired primary color is produced. The numbers in (**b**) are indicated in the color palettes in (**a**). The white arrows denote the *L̂* axis. **c** Corresponding locations of the mixed colors on the CIE 1931 Chromaticity Diagram. (i), (ii), and (iii) refer to the samples in (**b**). The red line indicates the sRGB color gamut. **d** (i) Experimental demonstration of the electrical tunability of the color pixels using the fabricated metasurface in (**b**-ii) along with the LC cell. The color is modulated from green through to gray, and finally to magenta upon the application of electric field. (ii) Faux-colored scanning electron microscope image of the metasurface showing the subpixel layout for the measured sample. The scale bar represents 1 µm

Finally, we investigate the electrical tuning of the ellipsoidal meta-atoms as active pixels in full-color reflective display applications. Mixing the reflectance spectra of the primary additive colors (red, green, and blue) allows the production of the secondary colors of yellow, magenta, and cyan, as well as white when all three are combined (Fig. 5a). Since the anisotropic meta-atoms can produce both bright colors and dark blacks, they can be combined to create pixels to display any shade and brightness of desired color. To prove this concept, we fabricate four separate samples. In each of the samples, the meta-atoms for one color are rotated by 90° compared to the other two, while for the final sample, all meta-atoms are aligned in the same direction. We observe all the designed colors, as well as dark black and bright white (Fig. 5b). The calculated locations of the mixed colors are plotted on the CIE 1931 Chromaticity Diagram, showing that even when mixed, the produced color gamut is comparable to the sRGB gamut. When the individual colors are combined into a single large pixel, the meta-atoms that are not required to contribute to the color production are necessarily black, therefore lowing the overall brightness of the colors compared to the individual subpixels. The brightness of the color is also affected by the size of the subpixels, with bigger subpixels providing brighter colors at the expense of larger pixels and lower PPI (Supplementary Note 11). Here, as a simple proof-of-concept, we combine the pixels in a 1:1:1 manner that produces a white pixel that has a warm tone. This is due to the individual red, green, and blue pixels reflecting different amounts of light. This could be compensated for by adjusting the size of each of the subpixels individually, so they contribute the appropriate amount of color to produce a white tone of the desired temperature (Supplementary Note 12). Horizontal lines of meta-atoms are chosen for the design of the subpixels, which maximizes the number of meta-atoms for the subpixels in the direction of the required momentum matching to induce the qGMR. As a compromise between resolution and brightness, we choose subpixels to have four horizontal lines of meta-atoms per color, resulting in a PPI of ~6000. By integrating the sample in Fig. 5b ii with the LC cell, electrical tuning of the color from green to magenta is achieved using the external bias (Fig. 5d). With the LP of the incident light on the LC cell set to LP 0^o^, as the LCs modulate *L̂*, the reflected color is tuned from green at *E* = 0.0 V µm^−1^ (where *L̂*∥*â* for green and *L̂*⊥*â* for red and blue), through to gray at *E* = ~0.4 V µm^−1^ (where *L̂* is at ~45° to *â* for all meta-atoms), finally to magenta at *E* = 1.0 V µm^−1^ (where *L̂*∥*â* for red and blue and *L̂*⊥*â* for green). It should be noted that the discrepancy between the magenta color produced using static polarizers and the LC cell is due to the LCs not providing the exact phase retardance required for complete conversion between orthogonal LP states at the applied biases, which is a technical issue rather than a fundamental flaw with the device design and fabrication.

## Conclusion

In conclusion, we have designed, characterized, and experimentally realized a platform for electrically tunable structural color using all-dielectric metasurfaces in combination with LCs. We first presented high resolution and contrast static color printing, furthermore, by employing LCs as a method of electrically controlling the LP of the incident light, we proved the use of the anisotropic meta-atoms for use in electrically tunable metasurfaces as cryptographic and reflective display applications. To the best of our knowledge, this is the first demonstration of fully controllable metasurface-based structural color that can produce not only bright saturated colors, but also black and white pixels, as well as all combinations of states in between. Furthermore, no extra polarizers are required to block the reflected light and produce the black states, as they are achieved purely by suppressing the back scattering through the meta-atom design. The switching time of the color depends solely on the LC cell, and is generally on the scale of milliseconds. Since the spectral modulation is achieved with a large contribution from qGMR, the reflected colors depend on the incident angle of the light and the correct colors are retained up to angles of around 10° (Supplementary Note 13). Several interconnected factors should be considered for the final choice for the number of meta-atoms that make up a subpixel, which dictate the brightness and color temperature, and in turn the PPI of the device. This choice could be tailored for the desired purpose, such as high resolution, or a specific color temperature. Reflective displays could also have an important role to play in the IoT future, where masses of information are being consumed daily and energy efficiency is paramount, especially for locations of limited power supply. This proposed scheme can easily be applied in transmission mode albeit with the use of a subtractive color palette (Supplementary Note 14)^52^. Transflective displays could also be an interesting option for energy efficient display technologies^53^, but have yet to be achieved using metasurfaces. Due to the dependence on the refractive index of the surrounding media on the momentum matching conditions required for the hybridization and enhancement of the Mie scattering and qGMR, the proposed metasurfaces display asymmetric transmission (Supplementary Note 15), making it difficult to achieve such transflective displays. However, this could be overcome with the utilization of an index matching layer and the proper design of meta-atoms. Furthermore, the unique property of blocking certain amount of light at specific wavelengths has potential to be applied in smart windows or mirrors that modify the transmitted or reflected colors and their relative brightness. Moreover, the all-dielectric nature of the devices presented here lead to the simple integration with CMOS processes for scalable fabrication. The achievable color gamut using the design concept established here could possibly be further improved by employing materials with no optical losses throughout the visible regime, or by optimizing the meta-atom design using machine learning or computational techniques^54–56^.

It should be noted that the polarization of the incident light could also be controlled using electrically tunable waveplates with rotatable polarizers, and this element is one of the main limiting factors on resolution and miniaturization. The use of an additional polarizer in previous systems result in a significant loss of the intensity of the incident light, as only the light with polarization parallel to the optic axis of the polarizer can pass, which quickly becomes significant if the light passes the polarizer multiple times. The light intensity loss in the LC system, however, is negligible, leading to a much more energy efficient device with brighter and more vivid colors. The LC acts solely as a phase retarder to the LP state of light, therefore any subsequent interactions with the LC, such as after reflection from the metasurface, act only to retard the phase back to the original state. Therefore, LCs have the potential for further miniaturization, allowing for extremely high-resolution displays using metasurfaces.

From a technical point of view, the PPI of state-of-the-art displays varies based on the size of the device. As the size of the screen increases, the PPI naturally decreases, which requires the user to utilize the display from a farther distance in order to see a clear image. If we consider a few practical examples of the latest high-end 4 K displays in consumer devices, 6.5 inch mobile displays have reached ~650 PPI, whereas 24 inch monitors generally achieve ~200 PPI, and large televisions over ~40 inches provide ~100 PPI. For the metasurfaces proposed here to achieve a comparable PPI to these state-of-the-art displays, we could afford to utilize subpixels made up of ~35, ~120, and ~250 rows of meta-atoms for mobile displays, monitors, and large televisions, respectively. This could be easily achieved using the metasurfaces proposed here as demonstrated in Supplementary Note 11. However, a key hurdle to overcome before realizing such metasurface-based displays comes from limitations in large-scale nanofabrication. Recently, advances in nanoimprinting techniques have suggested a potential answer^11^, in particular, roll-to-roll printing could achieve the continuous printing of metasurfaces. Resin embedded with high-*n* dielectric nanoparticles has been proven as an exciting material for such nanoimprinting methods, and has also been used in direct 3D printing using two-photon lithography.

## Materials and methods

### Reflection spectrum simulation

An in-house developed RCWA solver^57^ was used to calculate the reflection spectra of the meta-atoms, under normal and oblique incidence. All color calculations were performed using the open-source package, ‘Color’, in Python. The D50 illuminant and CIE 1931 2° standard observer were used throughout. The electric and magnetic field profiles were calculated using the finite-difference time-domain (FDTD) solver, Lumerical, Ansys, with periodic boundary conditions in the *x*- and *y*-directions, and perfectly matched layers in the *z*-direction. In minimum pixel size simulations, perfectly matched layers are used for all boundaries and the simulation size along the *x-* and *y-*directions are set to

$$\left( {x,y} \right) = \left( {1 + \left( {N_x,N_y} \right)} \right) \times P$$

### Multipole expansion

The multipole scattering cross-sections were calculated using the commercially available FEM software, COMSOL Multiphysics. The multipole moments are given as

$$D_\alpha ^{{{\mathrm{e}}}} = - \frac{1}{{i\omega }}\mathop {\int}\limits_V {{{{\mathrm{d}}}}^3{{{\mathbf{r}}}}} \left\{ {j_0\left( {kr} \right)J_\alpha + \frac{{k^2}}{2}\left[ {3\left( {{{{\mathbf{r}}}} \cdot {{{\mathbf{J}}}}} \right)r_\alpha - r^2J_\alpha } \right]\frac{{j_2\left( {kr} \right)}}{{\left( {kr} \right)^2}}} \right\},$$

$$D_\alpha ^{{{\mathrm{m}}}} = \frac{3}{2}\mathop {\int}\limits_V {{{{\mathrm{d}}}}^3{{{\mathbf{r}}}}} \left( {{{{\mathbf{r}}}} \times {{{\mathbf{J}}}}} \right)_\alpha \frac{{j_1\left( {kr} \right)}}{{kr}},$$

$$\begin{array}{ll}Q_{\alpha \beta }^{{{\mathrm{e}}}} = - \frac{3}{{2i\omega }}\mathop {\int}\limits_V {{{{\mathrm{d}}}}^3{{{\mathbf{r}}}}} \left\{ \left[ {r_\alpha J_\beta + r_\beta J_\alpha - \frac{2}{3}\left( {{{{\mathbf{r}}}} \cdot {{{\mathbf{J}}}}} \right)\delta _{\alpha \beta }} \right]\frac{{j_1\left( {kr} \right)}}{{kr}}\right.\\\left. \quad\qquad+ \frac{{2k^2}}{3}\left[ {5\left( {r \cdot J} \right)r_\alpha r_\beta - r^2\left( {r_\alpha J_\beta + r_\beta J_\alpha } \right) - r^2\left( {{{{\mathbf{r}}}} \cdot {{{\mathbf{J}}}}} \right)\delta _{\alpha \beta }} \right]\frac{{j_3\left( {kr} \right)}}{{\left( {kr} \right)^3}} \right\}{,}\end{array}$$

$$Q_{\alpha \beta }^{{{\mathrm{m}}}} = \frac{5}{2}\mathop {\int}\limits_V {{{{\mathrm{d}}}}^3{{{\mathbf{r}}}}} \left[ {r_\alpha \left( {{{{\mathbf{r}}}} \times {{{\mathbf{J}}}}} \right)_\beta + r_\beta \left( {{{{\mathbf{r}}}} \times {{{\mathbf{J}}}}} \right)_\alpha } \right]\frac{{j_2\left( {kr} \right)}}{{\left( {kr} \right)^2}}$$

where $D_\alpha ^{{{\mathrm{e}}}}$, $D_\alpha ^{{{\mathrm{m}}}}$, $Q_{\alpha \beta }^{{{\mathrm{e}}}}$, $Q_{\alpha \beta }^{{{\mathrm{m}}}}$ are electric dipole, magnetic dipole, electric quadrupole, magnetic quadrupole, respectively, α, β = x, y, z, ${{{\mathbf{J}}}} = - i\omega \left( {\varepsilon - \varepsilon _{{{\mathrm{h}}}}} \right){{{\mathbf{E}}}}$ is the induced current density, ε(x, ω) and εh are the permittivity of the scatterer and the host medium (air), and jn(z) is the spherical Bessel function. The radiation power is given as $P = \frac{1}{{{\it{\epsilon }}_h^2}}\frac{{k^4}}{{12\pi \eta }}\mathop {\sum}\nolimits_\alpha {\left| {D_\alpha ^{{{\mathrm{e}}}}} \right|^2} + \frac{1}{{{\it{\epsilon }}_h^2}}\frac{{k^6}}{{12\pi \eta }}\mathop {\sum}\nolimits_{\alpha \beta } {\left| {Q_{\alpha \beta }^{{{\mathrm{e}}}}} \right|^2} + \eta ^2\frac{{k^4}}{{12\pi \eta }}\mathop {\sum}\nolimits_\alpha {\left| {D_\alpha ^{{{\mathrm{m}}}}} \right|^2} + \eta ^2\frac{{k^6}}{{12\pi \eta }}\mathop {\sum}\nolimits_{\alpha \beta } {\left| {Q_{\alpha \beta }^{{{\mathrm{m}}}}} \right|^2}$, where η is the wave impedance of the host medium. The scattering cross-sections are given as the radiation power divided by the power flux of the incident planewave, $E_0^2/\left( {2\eta } \right)$.

### Metasurface fabrication

Metasurfaces were fabricated on a 500 μm-thick glass substrate. A 110 nm-thick layer of a-Si:H was deposited using plasma enhanced chemical vapor deposition (PECVD, BMR Technology HiDep-SC) with a flow rate of 10 sccm for SiH_4_ and 75 sccm for H2. Chamber pressure and operating temperature were 25 mTorr and 200 °C, respectively. The standard EBL process (ELONIX, ELS-7800) was used to transfer meta-atoms onto a single layer of positive tone photoresists (495 PMMA A2, MicroChem). Acceleration voltage and beam current is 80 kV and 100 pA, respectively. The exposed patterns were developed by MIBK/IPA 1:3 developer mixed solution. A 30 nm-thick chromium (Cr) layer was deposited using electron beam evaporation (KVT, KVE-ENS4004). The lift-off Cr meta-atoms were used as an etching mask for the a-Si:H film. Cr patterns were transferred onto the glass substrate using a dry etching process (DMS, silicon/metal hybrid etcher). The remaining Cr etching mask was removed by Cr etchant (CR-7).

### Patterned ITO glass plate preparation

A photoresist (AZ GXR-601, MERCK) was spin-coated at 1000 rpm for 10 s followed by at 2500 rpm for 30 s. Subsequently, the photoresist covered substrates were baked at 90 °C for 90 s. After UV exposure with a photomask for 20 s, the substrates were baked at 110 °C for 90 s. The exposed photoresist was removed using a developer (AZ 300 MF Developer, MERCK) for 1 min and etched in an acid solution (pure water:hydrochloric acid, 32–38% (Avantor Performance Materials, LLC):Nitric acid = 2:2:1) for 2.5 min.

### LC cell preparation

The LC cells were assembled using a patterned ITO glass plate coated with a polyimide (Nissan Chemical Korea) and a bare glass plate. Both plates were rubbed using velvet texture to achieve a unidirectional orientation of the LC molecules. The glass plates were assembled in the anti-parallel direction of the rubbing. The gap between the glass plates was set by glass spacers mixed with UV glue (NOA 65, Norland Products, Inc.). 5CB (Jiangsu Hecheng Display Technology Co., Ltd.) was filled into the cell in the isotropic state to avoid the memory effect of alignment by flow. The wires for applying the electric field were soldered onto the ITO patterns using lead after removing off the polyimide on the substrate.

### Optical measurement

A homebuilt optical measurement setup was used to image the realized colors and the reflection spectra. The source was a xenon arc lamp (Newport, 66907-150XF-R1). The light was collimated with two biconvex lenses (Thorlabs, LB1630) and passed through a linear polarizer (Thorlabs, LPVISC050), beam splitter (SIGMAKOKI, TFA-30C05-10), objective lens (Olympus, LMPLFNL 20X) and reflected from the sample. The reflected light passed the objective lens and reflected from the beam splitter. Finally, the light was captured by a CCD camera (Lumenera, INFINITY 2-1 RC), and was analyzed by a spectrometer (HORIBA, iHR 320).

## Supplementary information

Supplementary Information

Supplementary Video
