# Supplementary material for: Liquid crystal-powered Mie resonators for electrically tunable photorealistic color gradients and dark blacks
Source: Light Sci Appl. 2022 Apr 29;11:118. doi: 10.1038/s41377-022-00806-8 (PMC9054757; doi:10.1038/s41377-022-00806-8)
Supplement: Supplementary file 1 — Supplementary Information [file 41377_2022_806_MOESM1_ESM.pdf]

Supplementary Information for

Liquid crystal-powered Mie resonators for electrically tunable photorealistic color gradients and dark blacks

Trevon Badloe[1,†], Joohoon Kim[1,†], Inki Kim[1,2,3†], Won-Sik Kim[4,†], Wook Sung Kim[5], Young-Ki Kim[4,] Junsuk Rho[1,4,6,7]**

[1]Department of Mechanical Engineering, Pohang University of Science and Technology (POSTECH), Pohang 37673, Republic of Korea

[2]Department of Biophysics, Institute of Quantum Biophysics, Sungkyunkwan University, Suwon 16419, Republic of Korea

[3]Department of Intelligent Precision Healthcare Convergence, Sungkyunkwan University, Suwon 16419, Republic of Korea

[4]Department of Chemical Engineering, Pohang University of Science and Technology (POSTECH), Pohang 37673, Republic of Korea

[5]Department of Electrical Engineering, Pohang University of Science and Technology (POSTECH), Pohang 37673, Republic of Korea

[6]POSCO-POSTECH-RIST Convergence Research Center for Flat Optics and Metaphotonics, Pohang 37673, Republic of Korea

[7]National Institute of Nanomaterials Technology (NINT), Pohang 37673, Republic of Korea

† These authors contributed equally to this work
*Email: jsrho@postech.ac.kr, ykkim@postech.ac.kr

Supplementary Note 1: Comparison of tunable structural color metasurfaces with liquid crystals

Table S1. Comparison of experimentally reported tunable structural color metasurfaces with liquid crystals.

Mechanism	Type	Peak	Gamut	Color tunability	Black	Ref
Plasmonic	Transmission	80%	70% of sRGB	Blue to red to green	✗	S1
Plasmonic	Transmission	~80%	Unreported	Purple to yellow / cyan to magenta	✗	S2
Plasmonic	Transmission	~40%	Unreported	Switching between undefined colors	✗	S3
Plasmonic	Reflection	15%	'Large' CMY	CMY	✓*	S4
Plasmonic	Reflection	~50%	Comparable to high-quality print magazine	Blue to red to green	✗	S5
Plasmonic	Reflection	4% (scattering efficiency)	Comparable to sRGB	Red, green, and blue	✓*	S6
Plasmonic	Reflection	~50-80%	Unreported	Switching between undefined colors	✗	S7
**Dielectric**	**Reflection**	**~90% (R) ~60% (G) ~60% (B)**	**Comparable to sRGB**	**Continuous RGB to black**	✓	**This work**

**requires analyzer*

Supplementary Note 2: Measured refractive index of a-Si:H

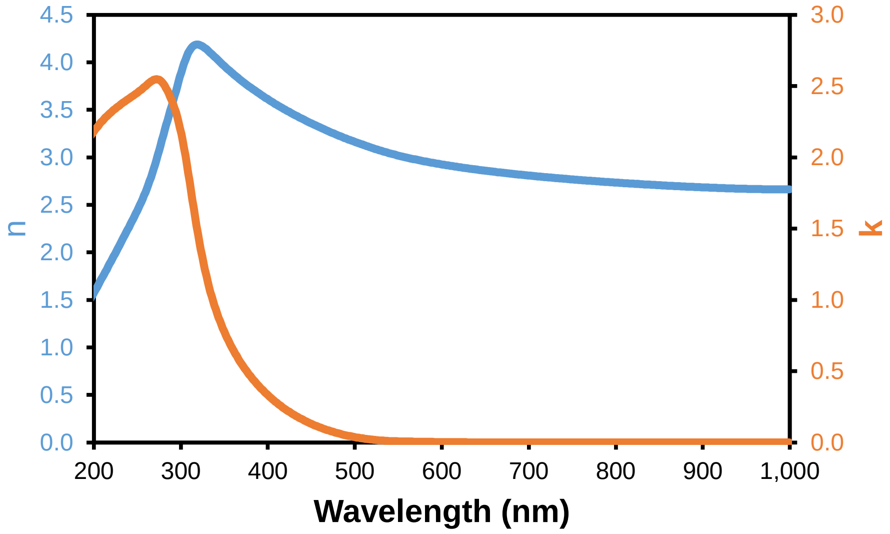

Figure S1. Measured refractive index of a-Si:H. The low-loss a-Si:H has a high refractive index (*n*), and low extinction coefficient (*k*) across the visible regime.

The high refractive index (*n*) and low extinction coefficient (*k*) of the a-Si:H lends well to applications in metasurfaces at visible wavelengths. The slightly higher *k* at blue wavelengths leads to some absorption and a lower reflectance when producing blue colors. This could be reduced further with a sacrifice of *n* by manipulating the deposition conditions of the material, [S8] but would also require the overall geometry of the meta-atoms to be optimized again.

Supplementary Note 3: Rectangular meta-atoms

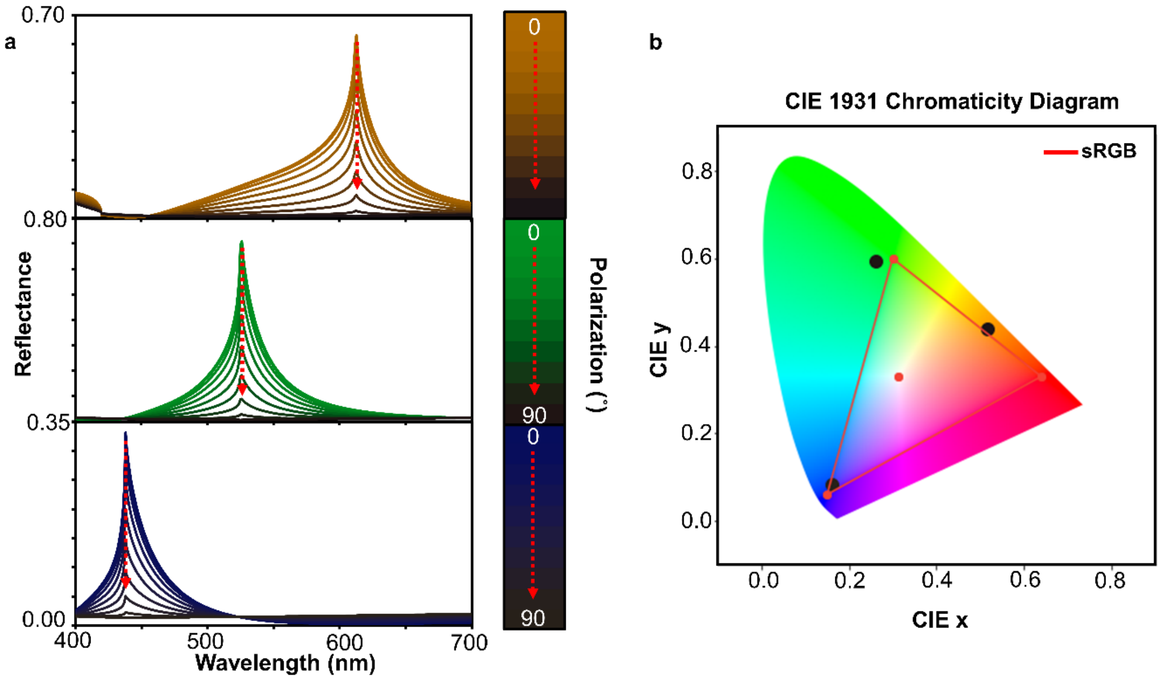

Figure S2. Rectangular meta-atoms. **(**a) Simulated reflection spectra of optimized meta-atoms for the primary additive colors, red, green, and blue. (b) CIE 1931 chromaticity diagram of the primary colors.

Other anisotropic shapes such as rectangular meta-atoms can also be used to produce the desired polarization dependency on the color, with high contrast with the dark blacks. However, for 110 nm high rectangular meta-atoms with the same long and short axis lengths as the elliptical meta-atoms in the main manuscript, the performance is significantly worse in terms in overall reflectance and in particular for red colors. It should be noted, nevertheless, the chosen dimensions were chosen to optimize elliptical meta-atoms, and there could indeed be dimensions that outperform the results shown here.

Supplementary Note 4: Excitation of quasi-guided mode resonance

As the array of nanoscale meta-atoms acts as a subwavelength grating, the light is diffracted in-plane at the Rayleigh-Wood anomaly (RA), which occurs at the wavelength at which the diffracted wave propagates at 90° to the grating.[S9] When the grating provides the required momentum to the incident light for evanescent wavevectors to match the in-plane lattice propagation vectors, quasi-guided mode resonances (qGMR) are produced. The wave vectors of the reciprocal lattice planes are determined by the grating period *P*, mode numbers *m* and *n* (defining diffraction orders in the *x*- and *y*-directions, respectively), and the incident wave vectors $k_{x,inc}$ *and* $k_{y,inc}$ by:

$$k_{x,mn} = k_{x,inc} - m\frac{2\pi}{P}, \tag{S1}$$

$$k_{y,mn} = k_{y,inc} - n\frac{2\pi}{P}. \tag{S2}$$

The propagation constant β of the leaky modes can be defined by the diffracted wave vectors as:

$$Re\{\beta\} = \sqrt{{k_{x,mn}}^2 + {k_{y,mn}}^2}$$
$$= \sqrt{k_{x,inc}^2 + k_{y,inc}^2 + \frac{4\pi}{P}(mk_{x,inc} + nk_{y,inc}) + (m^2 + n^2)\frac{4\pi^2}{P^2}} \tag{S3}$$

For the lattice modes to be allowed, the propagation constant must satisfy the relationship:

$$k_0 max[n_{sub}, n_{back}] \leq |Re\{\beta\}| < k_0 n_{eff}, \tag{S4}$$

where k_0 is the incident wavevector, and n_{sub}, n_{back}, and n_{eff} are the effective refractive index of the substrate, background medium, and the unit cell, respectively. At normal incidence ($k_{x,inc}$ = $k_{y,inc}$ = 0), so Equation 4 can be simplified to:

$$max[n_{sub}, n_{back}] \leq \lambda\left|\frac{\sqrt{m^2 + n^2}}{P}\right| < n_{eff} \tag{S5}$$

As an example, we numerically calculate the scattering cross-section of the meta-atom for producing green as a single meta-atom suspended in air (Figure S3a i), an infinite array of meta-atoms suspended in air (*n* = 1) (Figure S3a ii), and an infinite array of meta-atoms on a SiO_2 substrate (*n* = 1.46) (Figure S3a iii), both with *P* = 360 nm. All plots are normalized to the maximum scattering for ease of comparison. It is clear to see that the scattering cross-sections are enhanced through the hybridization of the qGMR from the periodic lattice and the Mie scattering from the individual meta-atoms. When the meta-atoms are arranged periodically in air, the location of the RA is at λ = 360 nm, to the blue side of the figure. However, when arranged on a substrate, the left side of Equation 5 is increased, and the RA is subsequently red shifted to around λ = 1.46×360 nm ≈ 525 nm, as highlighted in Figure S3c

with the gray dotted line. The electric and magnetic multipole resonances are therefore enhanced to the red side of this location.

Furthermore, we calculate the electric fields at the resonance locations for each setup, as shown in Figure S3b i-iii. The field profiles are similar when the meta-atoms are suspended in air or arranged on top of a substrate, highlighting the fact that the enhanced directional scattering can be largely attributed to the subwavelength grating produced by the array of meta-atoms which excites the qGMR.

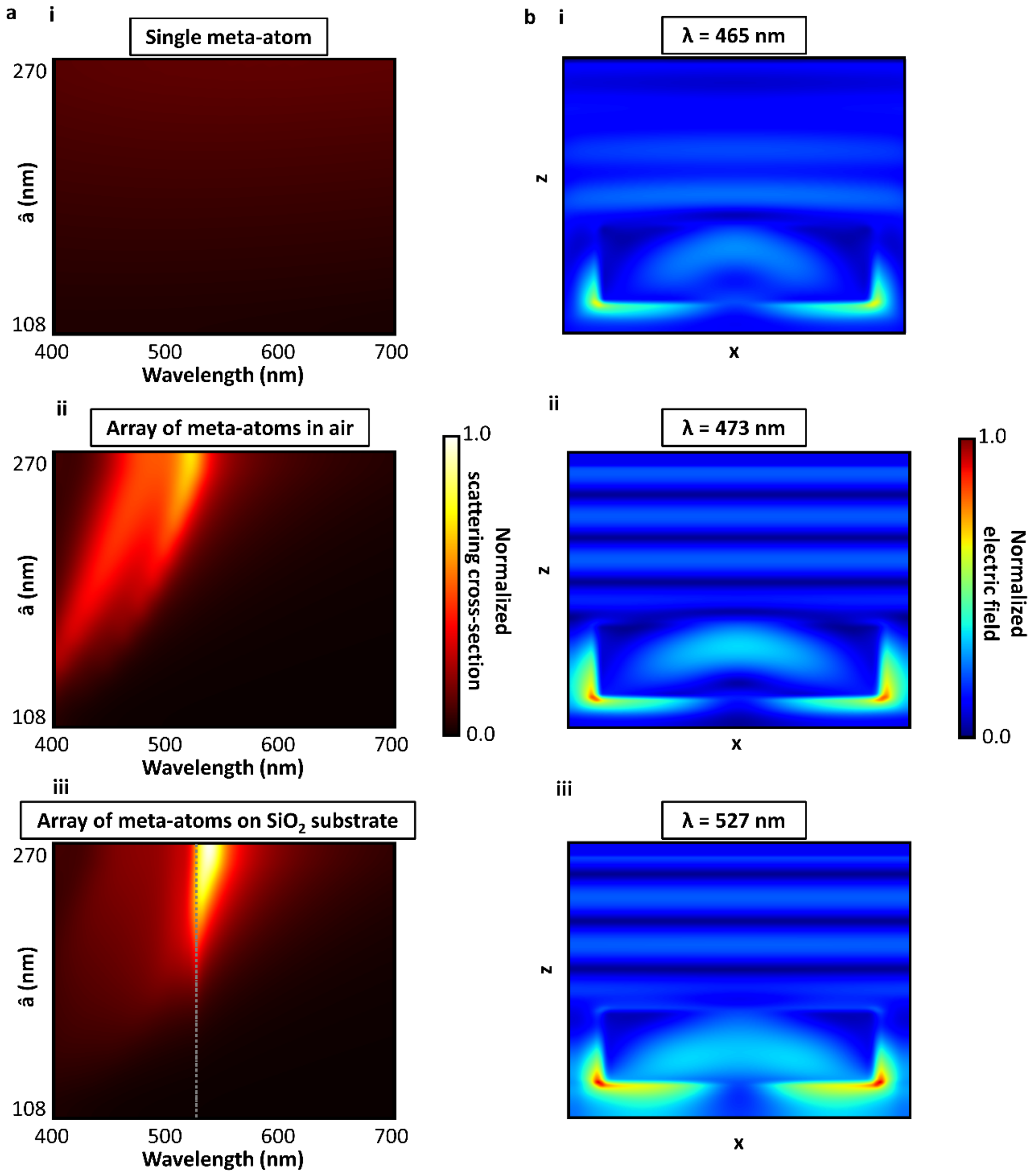

Figure S3. Effect of periodic arrangement of meta-atoms on the spectral modulation. (a) Simulated scattering cross-section of the green color producing meta-atom for (i) a single meta-atom in air, (ii) an infinite array of meta-atoms suspended in air (n = 1), and (iii) an infinite array of meta-atoms arranged on an SiO_2.substrate (*n* = 1.46). The dotted gray line

shows the location of the RA. (b) Calculated electric field profiles at the resonant locations of each setup in (a).

Supplementary Note 5: 140 nm height meta-atoms

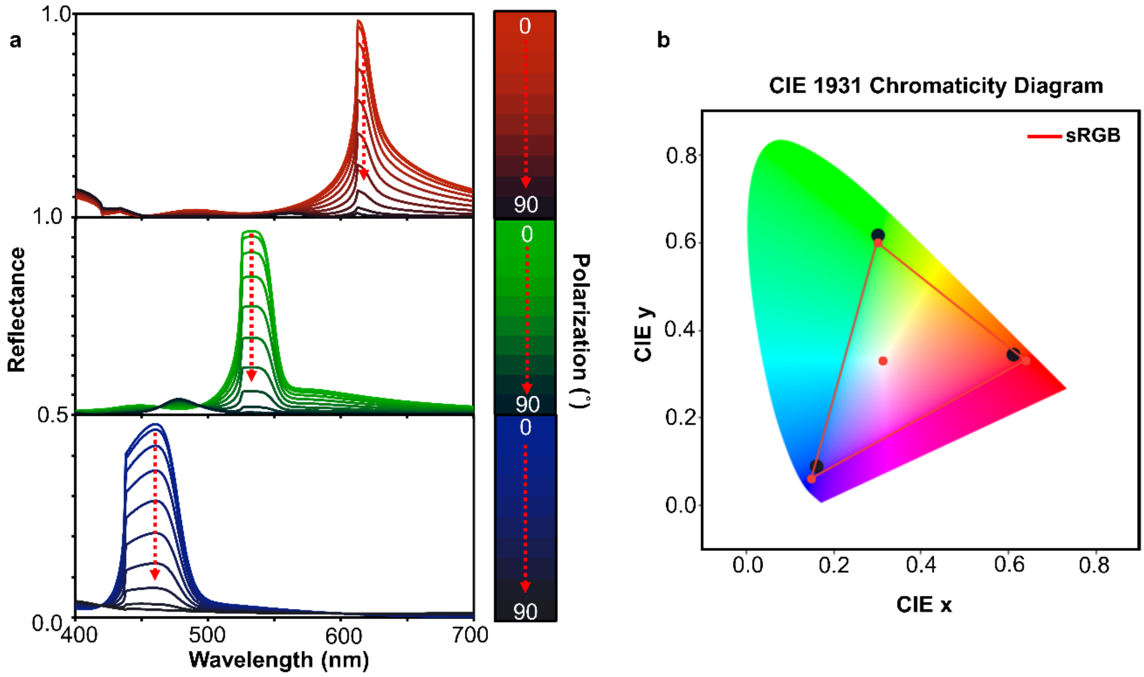

Figure S4. Performance of elliptical meta-atoms with a height of 140 nm. (a) Simulated reflection spectra of optimized meta-atoms for the primary additive colors, red, green, and blue. (b) CIE 1931 chromaticity diagram of the primary colors.

Compared to the meta-atoms presented in the main manuscript, slightly taller meta-atoms with a height of 140 nm produce bright colors that extend over a larger gamut on the CIE 1931 chromaticity diagram. However, for the green pixel in particular, it is difficult to produce a response with negligible scattering and reflection to make a black state. Color difference (ΔE) values of 11.4, 18.9, and 13.9 for red, green, and blue are achieved, however for the black states, ΔE values of 10.5, 14.3, and 7.6 are calculated. As black states are paramount for achieving colors that can be mixed with as little background reflectance as possible, the values of ΔE are insufficient.

Supplementary Note 6: Simulated color gamut

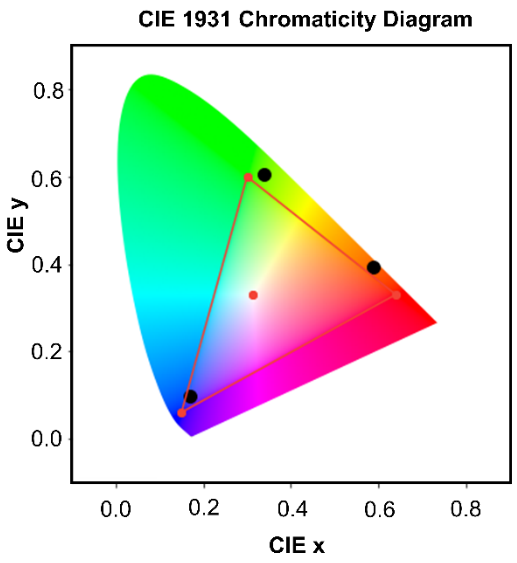

Figure S5. Simulated color gamut. The simulated color gamut of the red, green, and blue colors from the anisotropic meta-atoms plotted on the CIE 1931 Chromaticity Diagram. The red line indicates the sRGB color gamut, and the red dot denotes the standard white point.

Supplementary Note 7: Definition of color difference

Color difference (ΔE) is determined using the CIEDE2000 definition for colors converted from the CIELAB color space as,

$$\Delta E_{00}^{*} = \sqrt{\left(\frac{\Delta L'}{k_L S_L}\right)^2 + \left(\frac{\Delta C'}{k_C S_C}\right)^2 + \left(\frac{\Delta H'}{k_H S_H}\right)^2 + R_T \frac{\Delta C'}{k_C S_C}\frac{\Delta H'}{k_H S_H}} \quad \text{(S6)}$$

which considers compensation for a hue rotation (R_T), lightness (S_L), chroma (S_C), and hue (S_H) terms. k_L, k_C, and k_H terms are generally unity. A more detailed description can be found in the literature.[S10] All calculations were implemented through the open-source Python package 'Colour' (https://github.com/colour-science/colour).

Supplementary Note 8: Scattering cross-section dependence on incident polarization

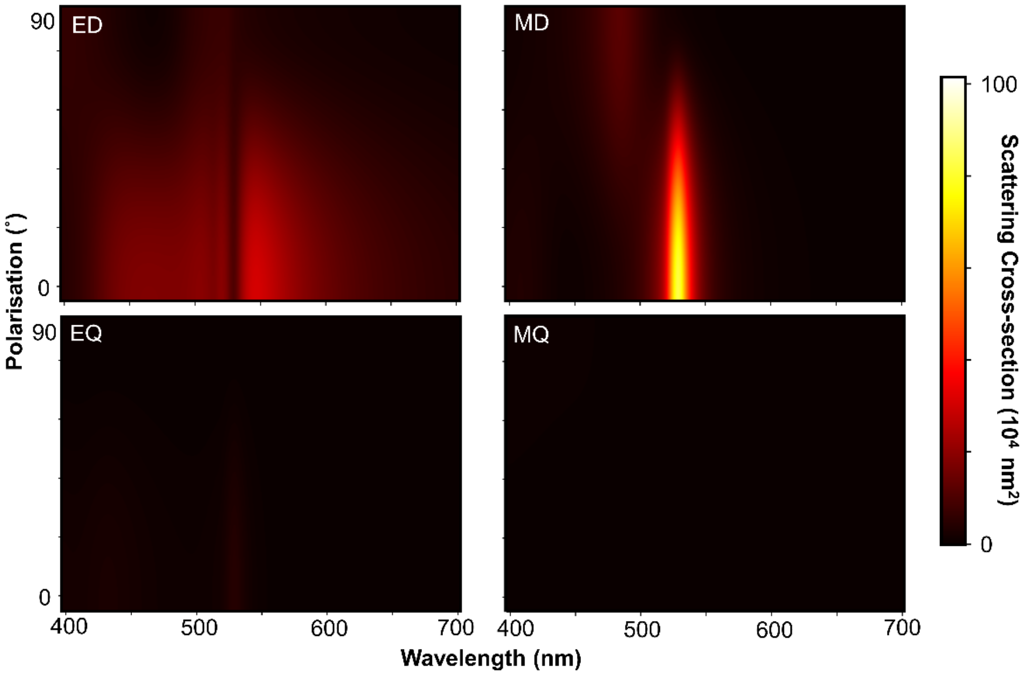

Figure S6. Scattering cross-section dependence on incident polarization. Heat maps of the scattering cross-section of the green producing metasurface, up to the quadrupole term.

From Figure S6, it is clear that the dominant scattering modes of the meta-atoms come from the MD, with a small contribution from the ED. The electric quadrupole (EQ) and magnetic quadrupole (MQ) are over a magnitude of power lower, so can be considered to be negligible in the overall scattering cross-section for all incident polarization. The spectral position of the MD is consistent for all incident polarizations, with a decrease in power and bandwidth as the incident angle is modulated from along the long to the short axis of the ellipsoid meta-atoms.

Supplementary Note 9: Color palette for different periodicities and consistent black states

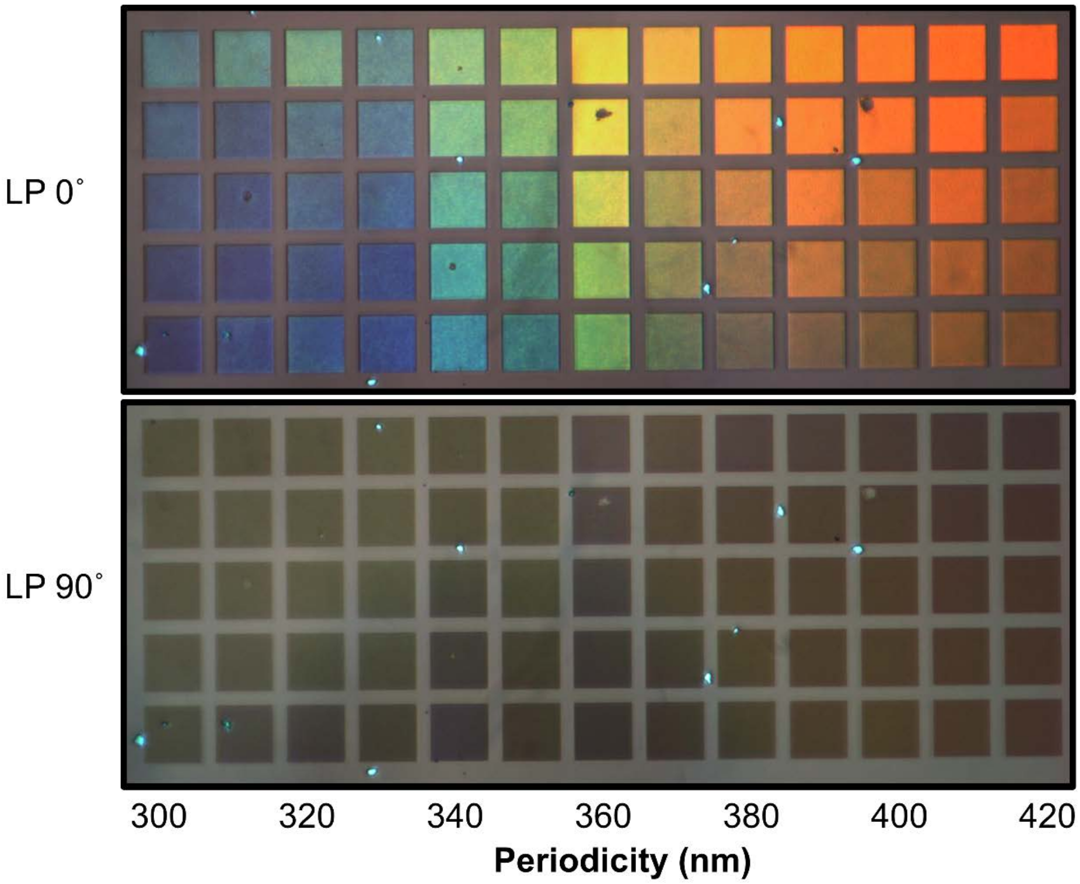

Figure S7. Experimentally realized color palette for various colors and consistent blacks with the same periodicity of meta-atoms. When the incident light polarization is LP 0˚, the bright colors are displayed, while they are suppressed to black under LP 90˚ illumination.

As the Mie scattering properties of the meta-atoms are defined by their overall geometry, various colors can be obtained for meta-atoms of the same periodicity, while the black states that require near-zero scattering can be conserved. This has potential uses for colorful cryptographic color prints. The geometric parameters of the meta-atoms are presented below in Table S2, with the simulated spectra and to-scale schematics of the green and blue meta-atoms are shown in Figure S8.

Table S2. Geometric parameters for meta-atoms with various hues of color with dark black states for the same periodicity.

***Period* (nm)**	**Meta-Atom #**									
	**1**		**2**		**3**		**4**		**5**	
	***a* (nm)**	***b* (nm)**	***a* (nm)**	***b* (nm)**	***a* (nm)**	***b* (nm)**	***a* (nm)**	***b* (nm)**	***a* (nm)**	***b* (nm)**
**300**	234	70	216	67	204	71	192	71	180	79
**310**	248	74	217	69	205	72	192	75	186	84
**320**	256	77	224	72	211	74	198	75	192	86
**330**	251	75	231	76	218	78	205	80	198	85
**340**	258	78	245	81	231	83	218	87	204	88
**350**	266	93	252	88	238	83	224	85	210	88
**360**	288	109	274	109	259	114	245	110	230	104
**370**	289	110	266	107	252	106	244	107	229	103
**380**	319	112	296	104	274	101	251	100	236	104
**390**	351	105	320	102	304	103	281	104	250	105
**400**	360	108	336	104	312	100	288	98	272	98
**410**	369	111	353	109	328	108	303	103	287	100
**420**	378	113	336	111	311	106	294	106	277	111

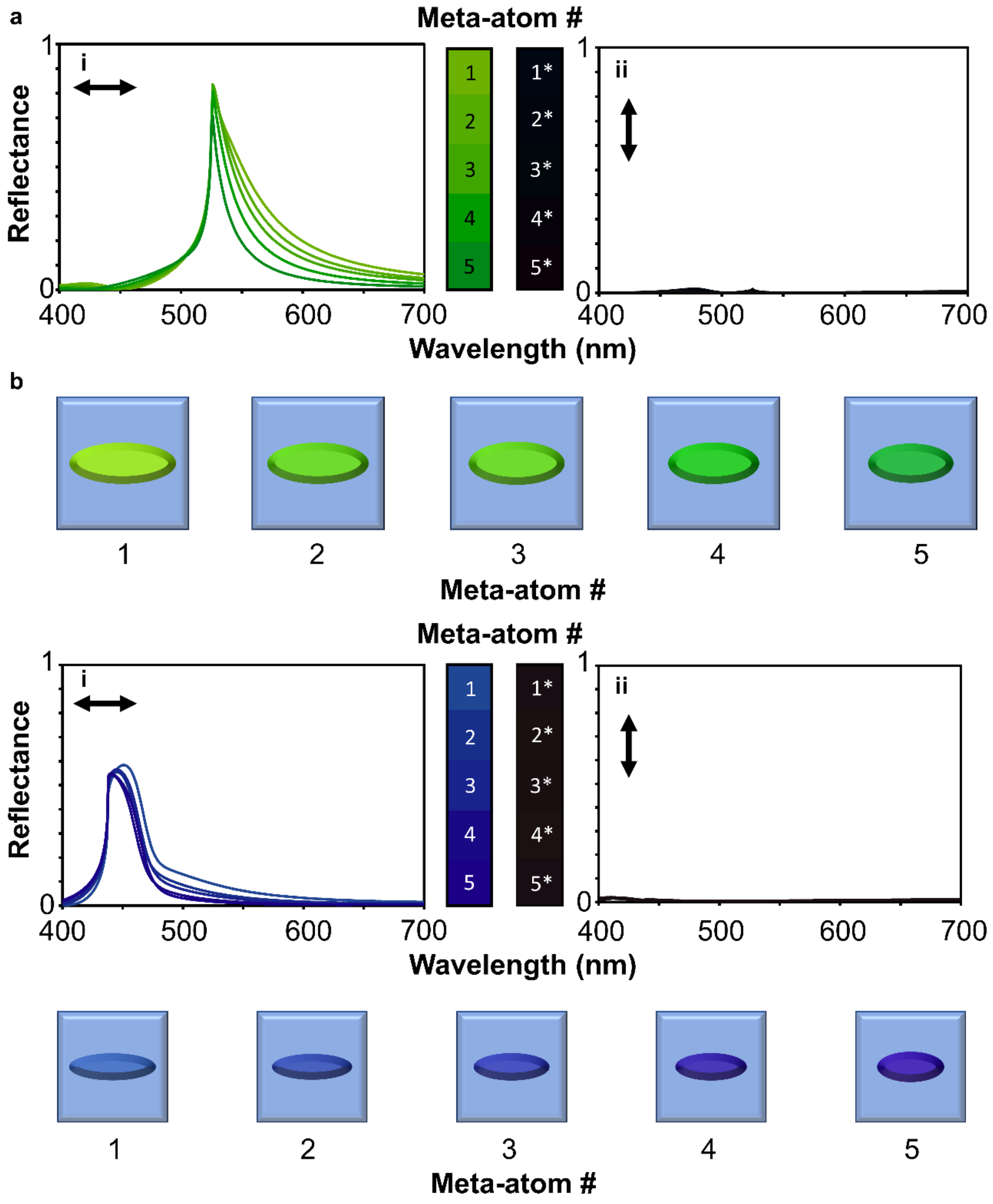

Figure S8. Selected meta-atoms for the cryptographic metasurfaces in the main manuscript. Simulated reflectance for LP 0° and LP 90° incident light (a) and (c) and to-scale illustrations of the corresponding meta-atoms (b) and (d) for green and blue colors.

Supplementary Note 10: Liquid crystal cell design and discussion

LCs exist in an intermediate phase between being a liquid and a crystal, exhibiting both molecular orientation and fluid-like properties. Exploiting these features enables the control the polarization of incident light though the application of an external stimulus to the LC cell.[S11] The polarization of the transmitted light is controlled by the retardation of the electromagnetic field that is induced by the LC system.[S12, S13] The retardation of the LCs (Γ) can be expressed as,

$$\Gamma = \Delta n \cdot d \quad \text{(S1)}$$

where Δn is the birefringence of the LCs and d is the thickness of the LC cell.[S14] The birefringence of the LCs is an inherent property of the chosen molecules and allows alteration of the refractive index of the cell by the orientation of the LC molecules. We employ 5CB(4-Cyano-4'-pentylbiphenyl) LCs, which have a Δn of around 0.818 at 25 ˚C.[S15] As the applied field on the LC cell is increased from 0 to 3 V μm^{-1}, the LC molecules align to electric field and convert the incident polarization, which in turn manipulates the brightness of the produced color through modulation of the Mie scattering response of the meta-atoms. With no applied electric field, the LC molecules are rotated by 135˚, due to unidirectional anchoring by rubbing both plates of a 5.4 μm thick sandwich cell, producing a fixed retardation of 3π radians. The polarization of the light is therefore completely shifted to the orthogonal state from the incident polarization when no bias is applied. At 0.5 V μm^{-1}, the direction of the LC molecules is about 158˚, therefore the polarization of the transmitted light is 44˚ linear polarization. At 3 V μm^{-1}, the LC molecules are aligned perfectly parallel to the incident light, so the polarization of the light transmitted through the LC cell is unchanged.

Supplementary Note 11: Number of structures per subpixel

The simulated reflection spectra for an ideal metasurface considers an infinite array when using periodic boundary conditions. However, long range lattice effects play a key role, so finite sized metasurfaces present slightly different reflection spectra. The color of finite metasurfaces with $Nx \times Ny$ meta-atoms in the x- and y-directions from 2×2 to 32×32 are shown in Figure S9. As more meta-atoms are added, the reflection spectra tend toward the infinitely periodic meta-atom spectra for each color. With even as few as 4×4 the desired color is produced, albeit with a much lower amplitude.

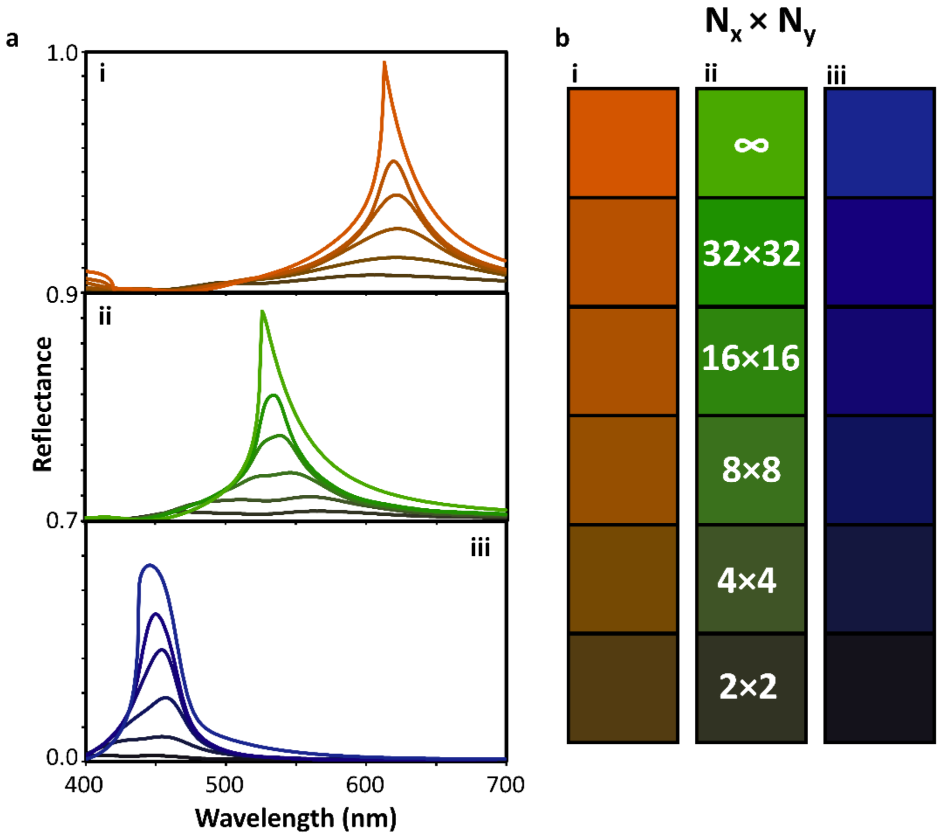

Figure S9. Effect of finite sized metasurfaces. (a) Simulated reflectance spectra and (b) colors for the (i) red, (ii) green, and (iii) blue metasurfaces with $N_x \times N_y$ structures in the x- and y-directions, respectively.

Considering the layout for the combined pixel, we investigate how the color is affected by the number of meta-atoms in the x- (Figure S10) or y-direction (Figure S11) separately. This is done using periodic boundary conditions in one direction. With an infinite number of meta-atoms in the x-direction, fewer meta-atoms in the y-direction produce a brighter color, closer to that of an infinite array. Therefore, we choose to combine the meta-atoms for color mixing with 4 lines of horizontal arrays of meta-atoms across the whole metasurface, as a compromise between color and resolution.

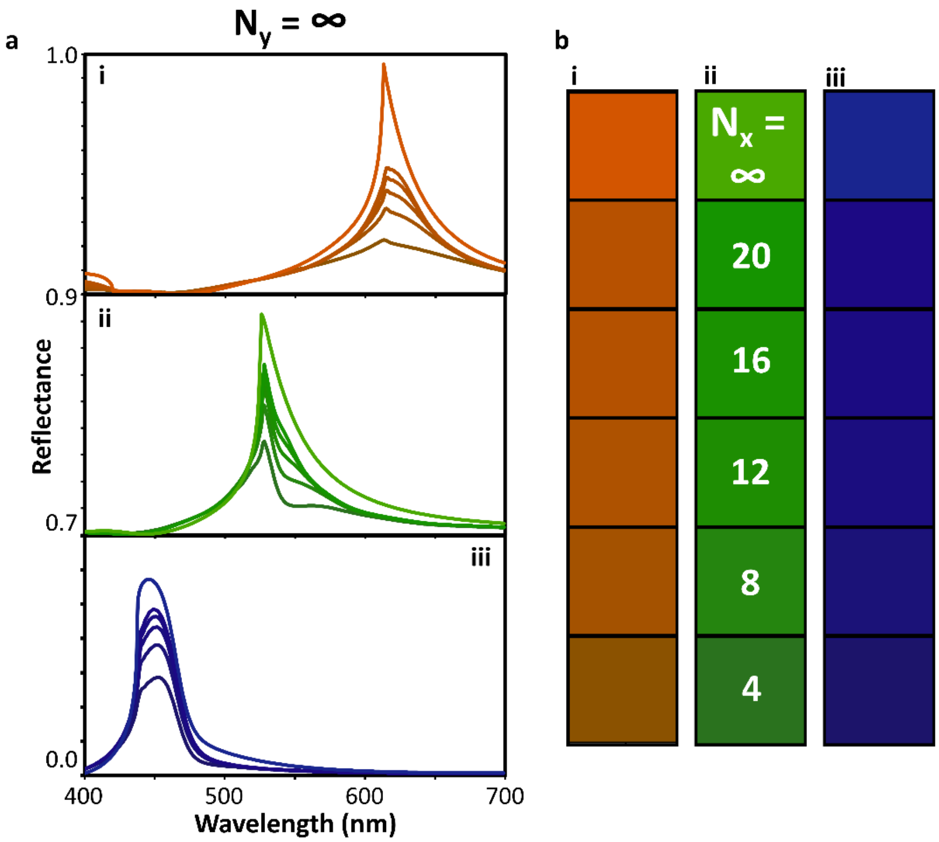

Figure S10. Effect of finite sized metasurfaces in the *x*-direction. (a) Simulated reflectance spectra and (b) colors for the (i) red, (ii) green, and (iii) blue metasurfaces with $N_y = \infty$, and N_x structures in the *y*- and *x*-directions, respectively.

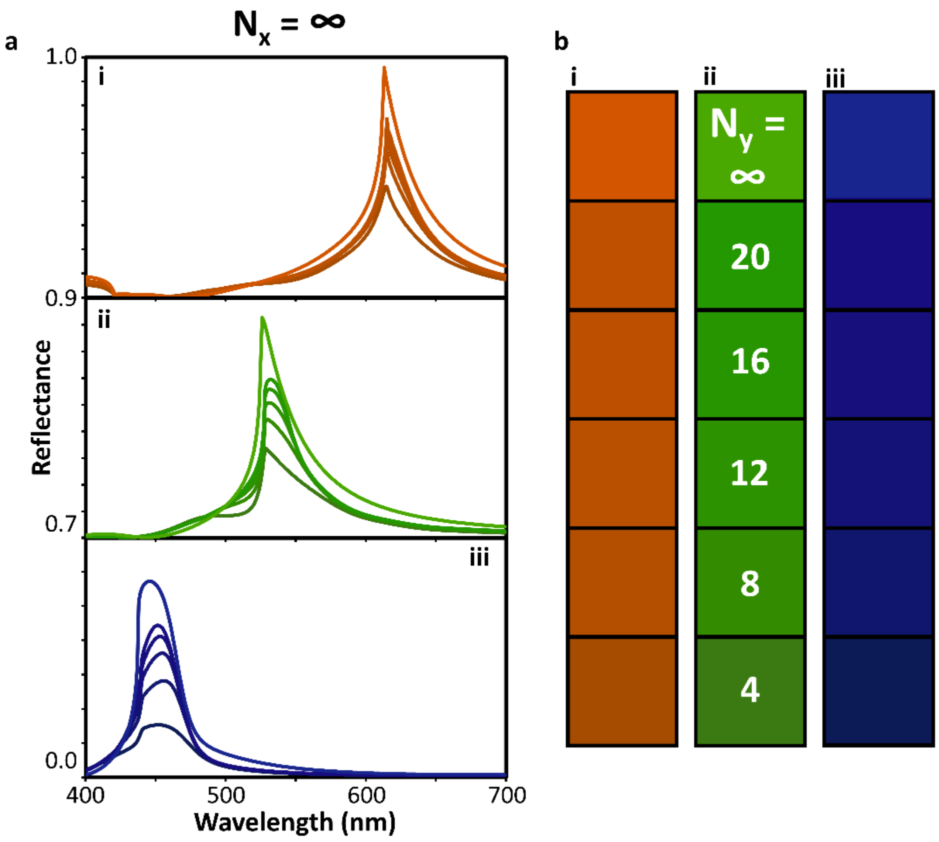

Figure S11. Effect of finite sized metasurfaces in the *y*-direction. (a) Simulated reflectance spectra and (b) colors for the (i) red, (ii) green, and (iii) blue metasurfaces with $N_x = \infty$, and N_y structures in the *x*- and *y*-directions, respectively.

Furthermore, we experimentally confirm the increase of brightness and decrease of pixel size with two samples, one with 4 meta-atoms per color in the *y*-direction, and one with 8 (Figure S12).

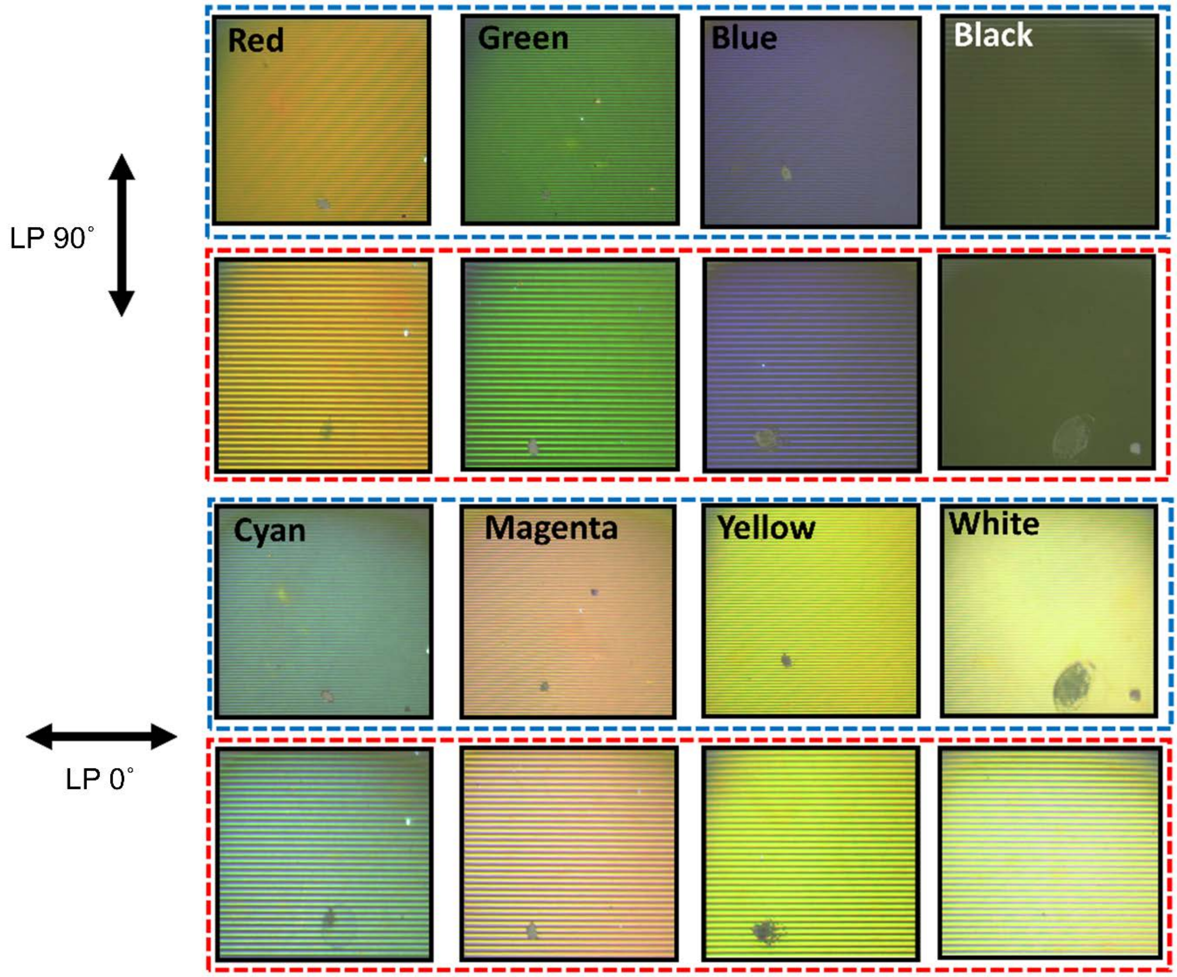

Figure S12. Effect of number of structures per subpixel. The pixels as displayed in the main manuscript with 4 subpixels in the vertical direction (blue dashed boxes), and the additional sample with 8 subpixels in the vertical direction (red dashed boxes). The incident polarization is demonstrated by the black arrow.

As the number of subpixels for each color in the pixel increases, for images at the same magnification, the colors appear brighter. However, the subpixels that display black become more visible and the minimum pixel size is increased. The overall reflection is lower than that of the individual subpixels since some part of the whole pixel does not participate in the color production when the incident polarization is along the short axis of that colors meta-atoms.

Supplementary Note 12: White point of the RGB pixels

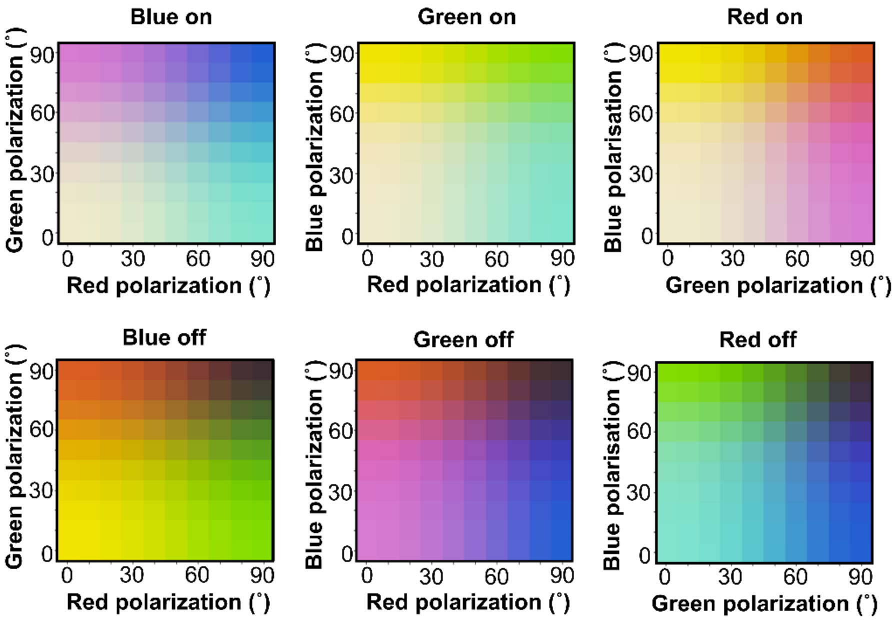

Figure S13. Mixed color palette for different white points. Color palettes with red, green, blue subpixel ratio of 1:2:4.

By combining the number of red, green, and blue subpixels with different ratios, the overall color palettes can be modulated. This can be understood through the temperature of the white point. For the ratio of RGB of 1:1:1 as demonstrated in the main manuscript, and Figure S12, the white point is a warmer tone, pushing all the colors to be more towards the warm, red side. By adding a larger ratio of blue subpixels to outweigh their lower total reflectance, more neutral white temperatures and colors could be achieved (Figure S13 and S14).

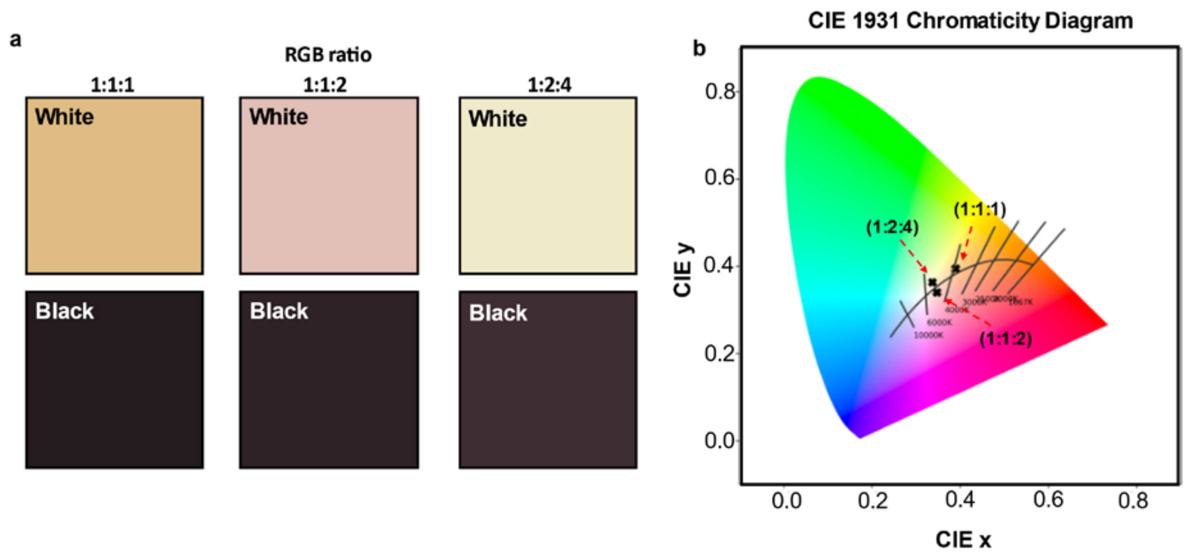

Figure S14. White temperature for different mixing ratios of the primary colors. The labelled points show the location of the white point for the relevant mixing ratios on the CIE 1931 chromaticity diagram.

Mixing the measured reflection responses from the fabricated samples with the ratios as indicated leads to different temperatures of white. Mixing with a 1:1:1 ratio leads to a white point at around 4,000 K, which appears slightly warm white, or orange color. As the ratio of blue and green are increased, the white becomes a cooler tone, around 5,000 K. Unfortunately, however, the black state of the blue and green pixels also slightly increases the brightness of the black state of the combined pixels.

Supplementary Note 13: Oblique Angle of Incidence

We numerically investigate the angular dependance of the designed structural color metasurfaces (Figure S15). It is clear to see that there is a strong interplay between the Mie scattering of the meta-atoms and the lattice effects due to the subwavelength grating induced from the periodic arrangement of the meta-atoms. The locations of the resonances strongly follow the regions where new diffractive modes arise. For both LP 0˚ and LP 90˚ the designed color or black is maintained for incident angle of up to around 10˚ of oblique incidence.

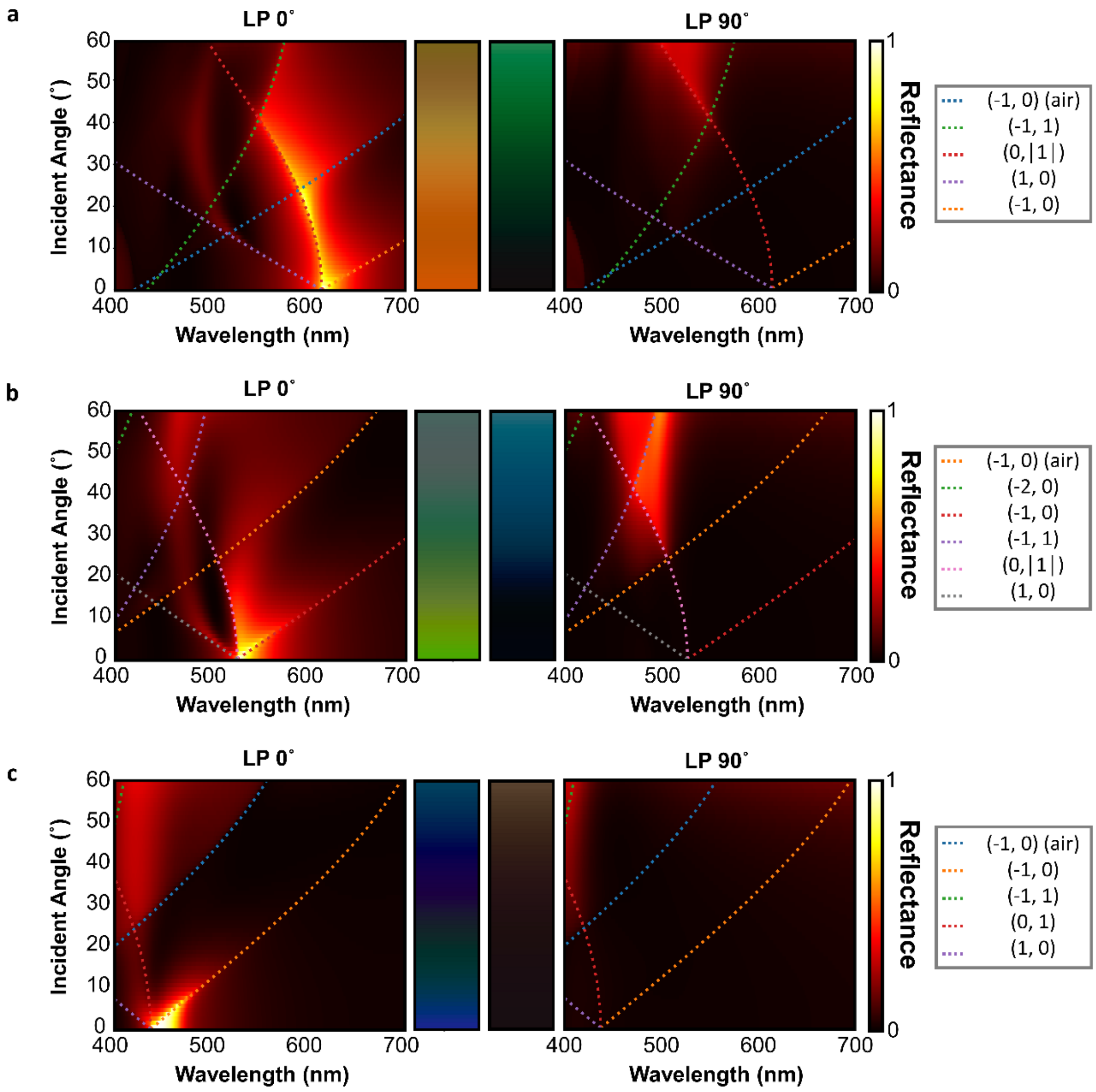

Figure S15. Oblique incident angle dependance. Heat maps of the reflectance of the designed metasurfaces under oblique incident illumination for LP 0˚ and LP 90˚ polarization of the (a) red, (b) green, and (c) blue metasurfaces. The reflected colors are presented in the bars in the center. The dotted lines represent the resonance locations of the *(m, n)* diffraction modes for $k_{y,inc} = 0$.

Supplementary Note 14: Transmission mode

The reflective color metasurfaces could alternatively be used in transmission mode (Figure S16). Rather than the additive color palette of red, green, and blue, however, the subtractive color palette of yellow, magenta, and cyan is produced, along with white rather than black. Due to the absorption from the a-Si:H at blue wavelengths, the white color under LP 90˚ has an unavoidable yellow tone. This could be potentially eliminated through the use of materials that are completely transparent across the whole visible regime, such as gallium nitride or titanium dioxide.

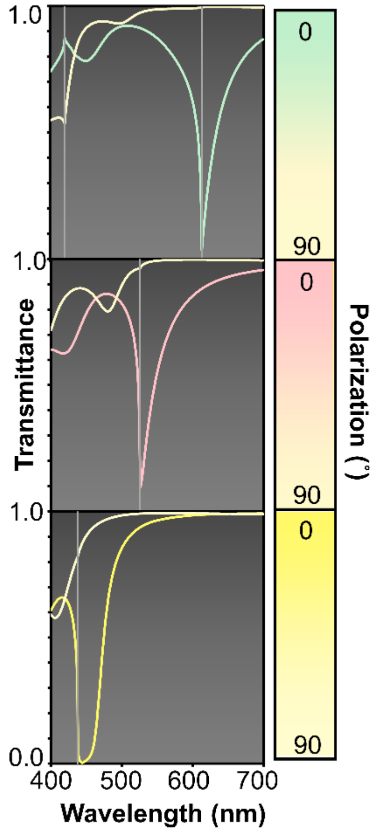

Figure S16. Transmission mode structural color. The red, green, and blue metasurfaces produce cyan, magenta, and yellow colors, respectively, in transmission for LP 0˚, and white instead of black under LP 90˚. The gray lines indicate the locations of the RA. For the cyan metasurface, there is an addition RA at 420 nm due to the background of air.

Supplementary Note 15: Asymmetric reflection and transmission

When the direction of the incident light is reversed, i.e., incident from the substrate to air, the spectral modulation is asymmetric (Figure S17). This could be problematic for applications such as transflective displays. However, index matching layers encapsulating the metasurfaces could be used to produce symmetric reflective and transmission, and could further protect the meta-atoms from physical damage.

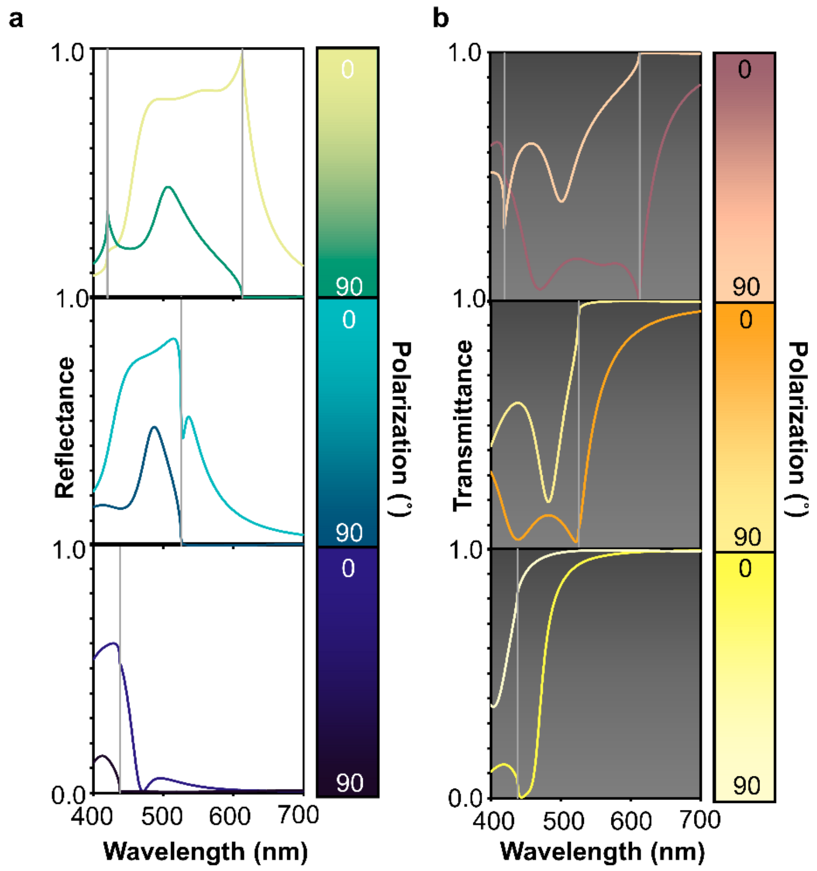

Figure S17. Asymmetric reflection and transmission of the metasurfaces. The red, green, and blue metasurfaces produce different colors in both (a) reflection and (b) transmission when the direction of incidence is reversed (from the SiO_2 substrate to air). The gray lines indicate the locations of the RA. For the red metasurface, there is an addition RA at 420 nm due to the background of air.

References

S1. Driencourt, L. *et al.* Electrically Tunable Multicolored Filter Using Birefringent Plasmonic Resonators and Liquid Crystals. *ACS Photonics* **7**, 444–453 (2020).

S2. Sharma, M., Hendler, N. & Ellenbogen, T. Electrically Switchable Color Tags Based on Active Liquid-Crystal Plasmonic Metasurface Platform. *Adv. Opt. Mater.* **8**, 1901182 (2020).

S3. Lee, Y. *et al.* Electrical Broad Tuning of Plasmonic Color Filter Employing an Asymmetric-Lattice Nanohole Array of Metasurface Controlled by Polarization Rotator. *ACS Photonics* **4**, 1954–1966 (2017).

S4. Franklin, D. *et al.* Self-assembled plasmonics for angle-independent structural color displays with actively addressed black states. *Proc. Natl. Acad. Sci.* **117**, 13350–13358 (2020).

S5. Franklin, D., Frank, R., Wu, S.-T. & Chanda, D. Actively addressed single pixel full-colour plasmonic display. *Nat. Commun.* **8**, 15209 (2017).

S6. Olson, J. *et al.* High chromaticity aluminum plasmonic pixels for active liquid crystal displays. *ACS Nano* **10**, 1108–1117 (2016).

S7. Franklin, D. *et al.* Polarization-independent actively tunable colour generation on imprinted plasmonic surfaces. *Nat. Commun.* **6**, 7337 (2015).

S8. Yang, Y. *et al.* Revealing Structural Disorder in Hydrogenated Amorphous Silicon for a Low-Loss Photonic Platform at Visible Frequencies. *Adv. Mater.* **33**, 2005893 (2021).

S9. Jang, J. *et al.* Spectral Modulation through the Hybridization of Mie-Scatterers and Quasi-Guided Mode Resonances: Realizing Full and Gradients of Structural Color. *ACS Nano* **14**, 15317–15326 (2020).

S10. Sharma, G., Wu, W. & Dalal, E. N. The CIEDE2000 color-difference formula: Implementation notes, supplementary test data, and mathematical observations. *Color Res. Appl.* **30**, 21–30 (2005).

S11. Kim, I. *et al.* Stimuli-Responsive Dynamic Metaholographic Displays with Designer Liquid Crystal Modulators. *Adv. Mater.* **32**, 2004664 (2020).

S12. *Soft Matter Physics: An Introduction*. (Springer New York, 2004). doi:10.1007/b97416.

S13. Yang, D. & Wu, S. *Fundamentals of Liquid Crystal Devices*. (Wiley, 2014). doi:10.1002/9781118751992.

S14. *Handbook of Liquid Crystals*. (Wiley-VCH Verlag GmbH & Co. KGaA, 2014). doi:10.1002/9783527671403.

S15. Li, J., Gauza, S. & Wu, S.-T. Temperature effect on liquid crystal refractive indices. *J. Appl. Phys.* **96**, 19–24 (2004).
